# Supplementary material for: Chiral expression from molecular to macroscopic level via pH modulation in terbium coordination polymers
Source: Nat Commun. 2017 Dec 14;8:2131. doi: 10.1038/s41467-017-02260-2 (PMC5730601; doi:10.1038/s41467-017-02260-2)
Supplement: Supplementary file 1 — Supplementary Information [file 41467_2017_2260_MOESM1_ESM.pdf]

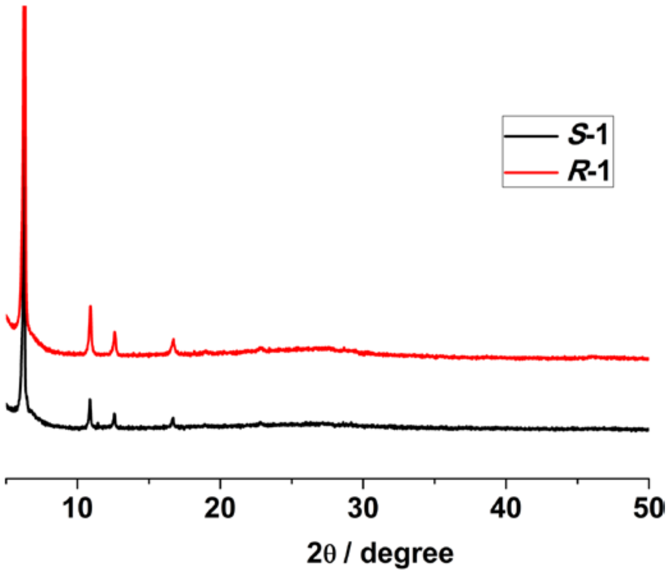

Supplementary Figure 1 | PXRD patterns of helices of ***R*-1** and ***S*-1**.

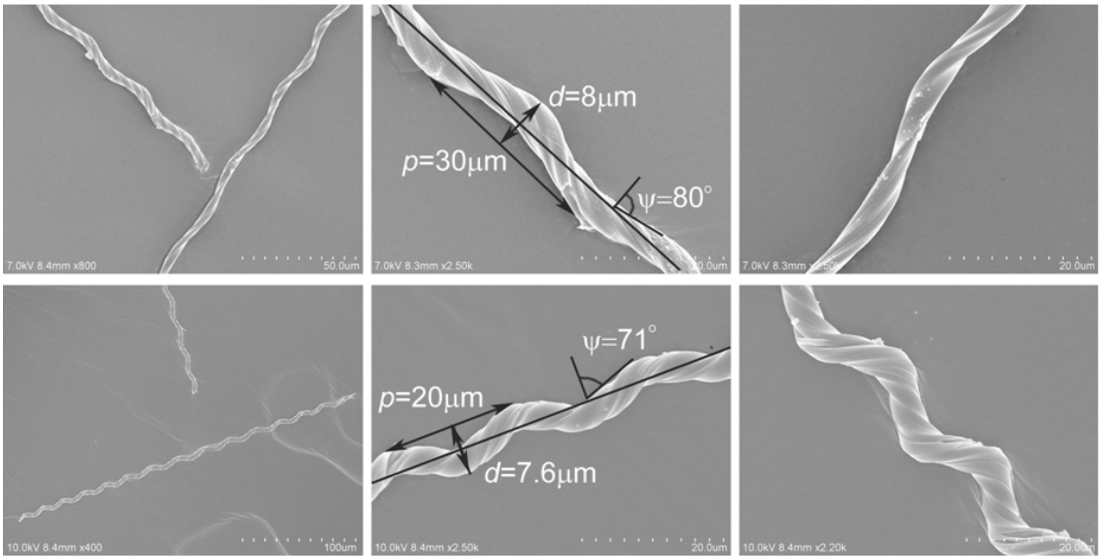

d: diameter *p*: pitch ψ: pitch angle

Supplementary Figure 2 | Pitch vs. diameter for helices. Pitch angles were found to be 70 ~ 80° for most helices. (top: right-handed helices of ***R*-1**; bottom: left-handed helices of ***S*-1**).

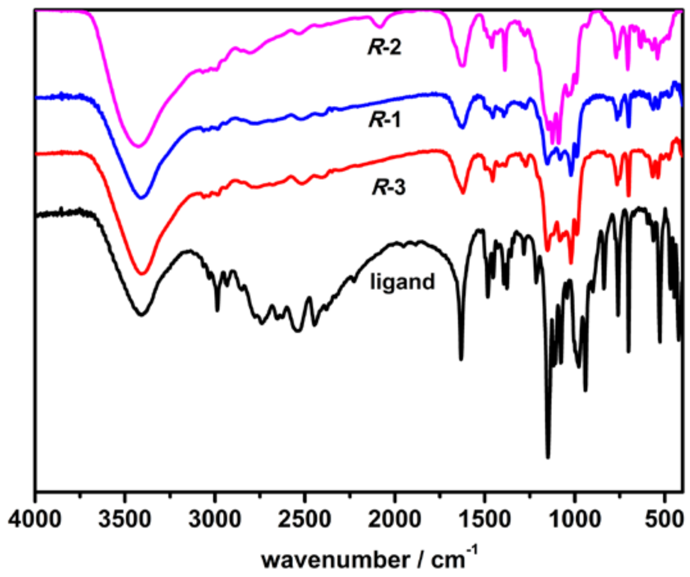

Supplementary Figure 3 | IR spectra of *R*-pempH_2, block-like crystals of ***R*-2** and rod-like crystals of ***R*-3**, and the helices of ***R*-1**.

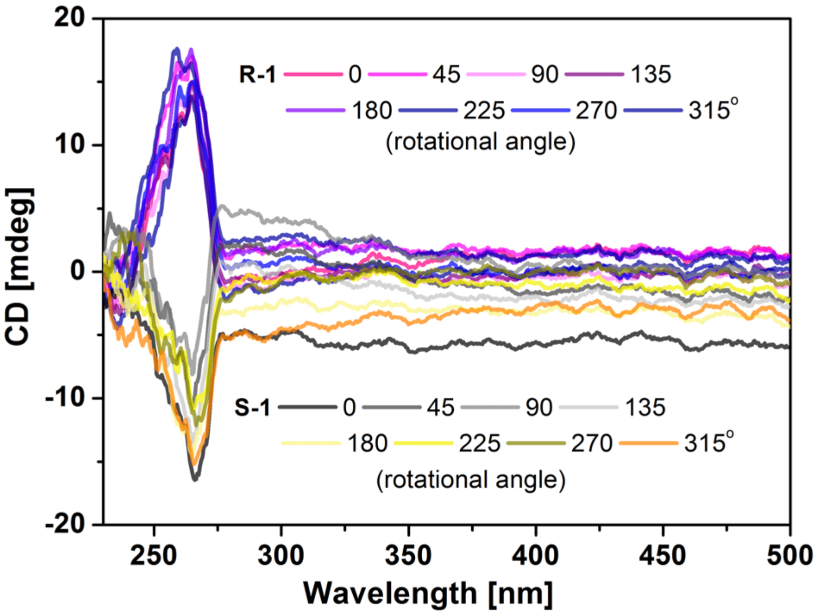

Supplementary Figure 4 | The angle dependence CD spectra of ***R*-1** and ***S*-1**.

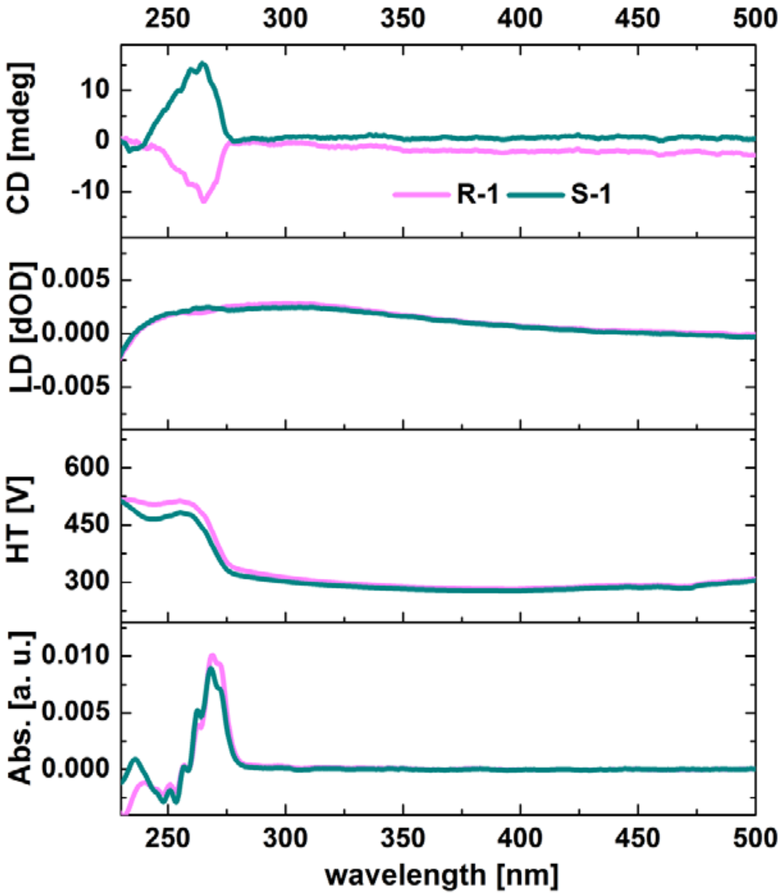

Supplementary Figure 5 | The average CD, LD, HT and absorption spectra of ***R*-1** and ***S*-1**.

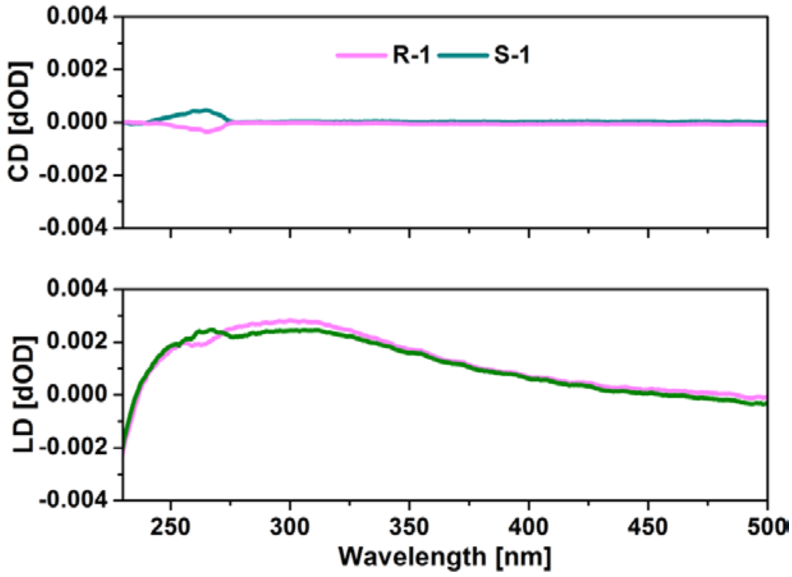

Supplementary Figure 6 | The comparison of CD and LD spectra in the same [dOD] unit for ***R*-1** and ***S*-1**. The peak height of CD signals is ca. 3 times that of LD signals, confirming that the observed CD spectra are true.

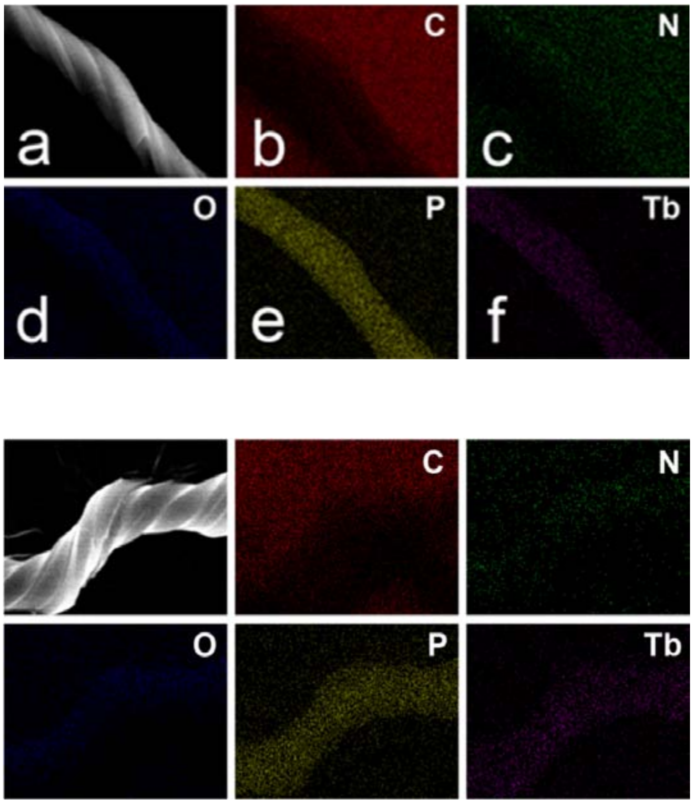

Supplementary Figure 7 | EDX map of ***R*-1** (top) and ***S*-1** helices (bottom).

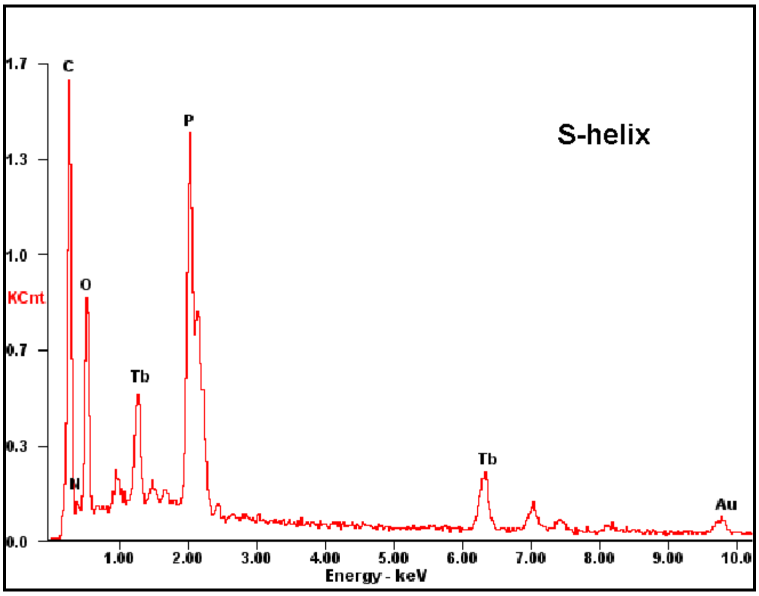

Supplementary Figure 8 | EDX spectra of ***R*-1** and ***S*-1** helices.

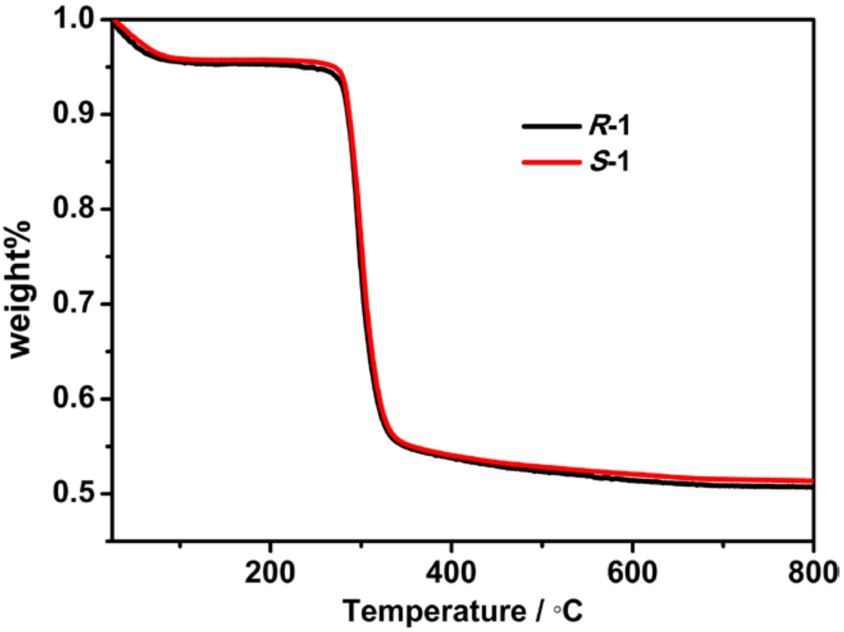

Supplementary Figure 9 | TGA curves of helices of ***R*-1** and ***S*-1**.

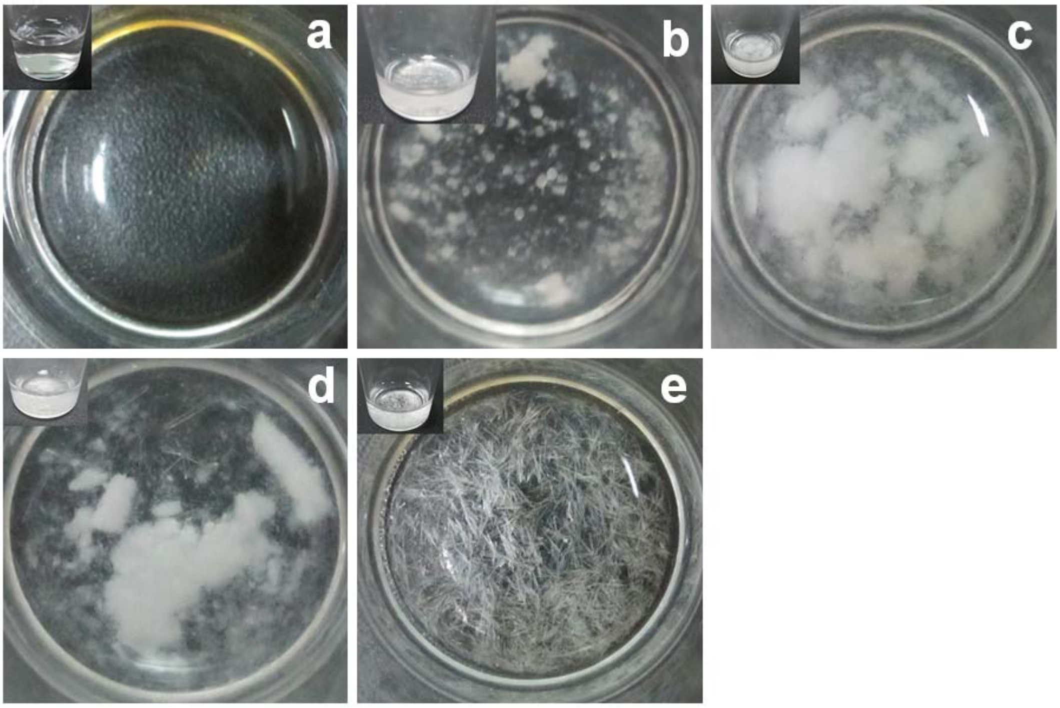

Supplementary Figure 10 | Photographs of the products after hydrothermal reactions of $Tb(NO_3)_3$ and *R*-pempH_2 at 120 °C for 2 d at different pH values. (a) pH < 2.5: Clear solution without any precipitates. (b) pH 2.5-2.9: Block-like crystals and white precipitates. (c) pH 3.0-3.2: White precipitates of helices. (d) pH 3.3-3.6: Mixture of helices and rod-like crystals. (e) pH 3.7-4.5: Colourless rod-like crystals.

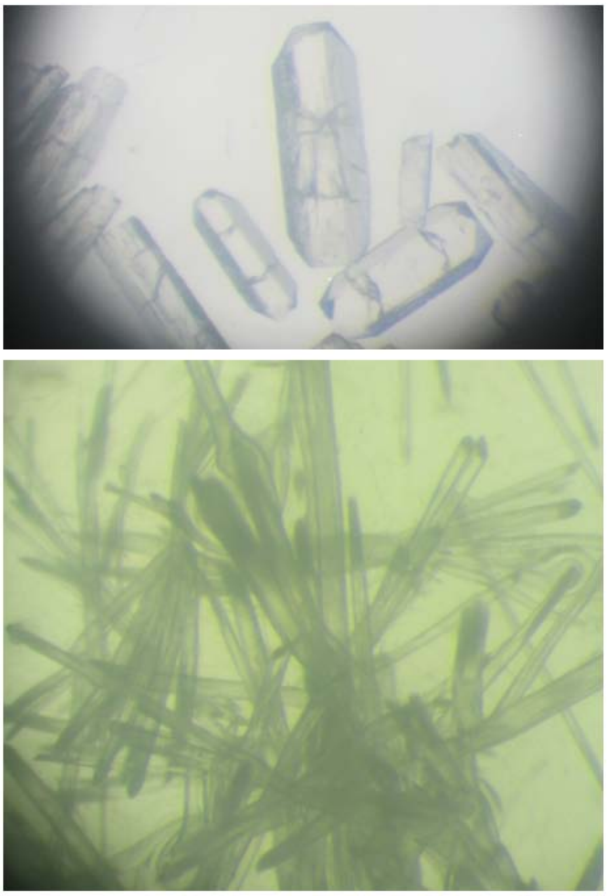

Supplementary Figure 11 | Photographs of block-like crystals of ***R*-2** (top) and rod-like crystals of ***R*-3** (bottom).

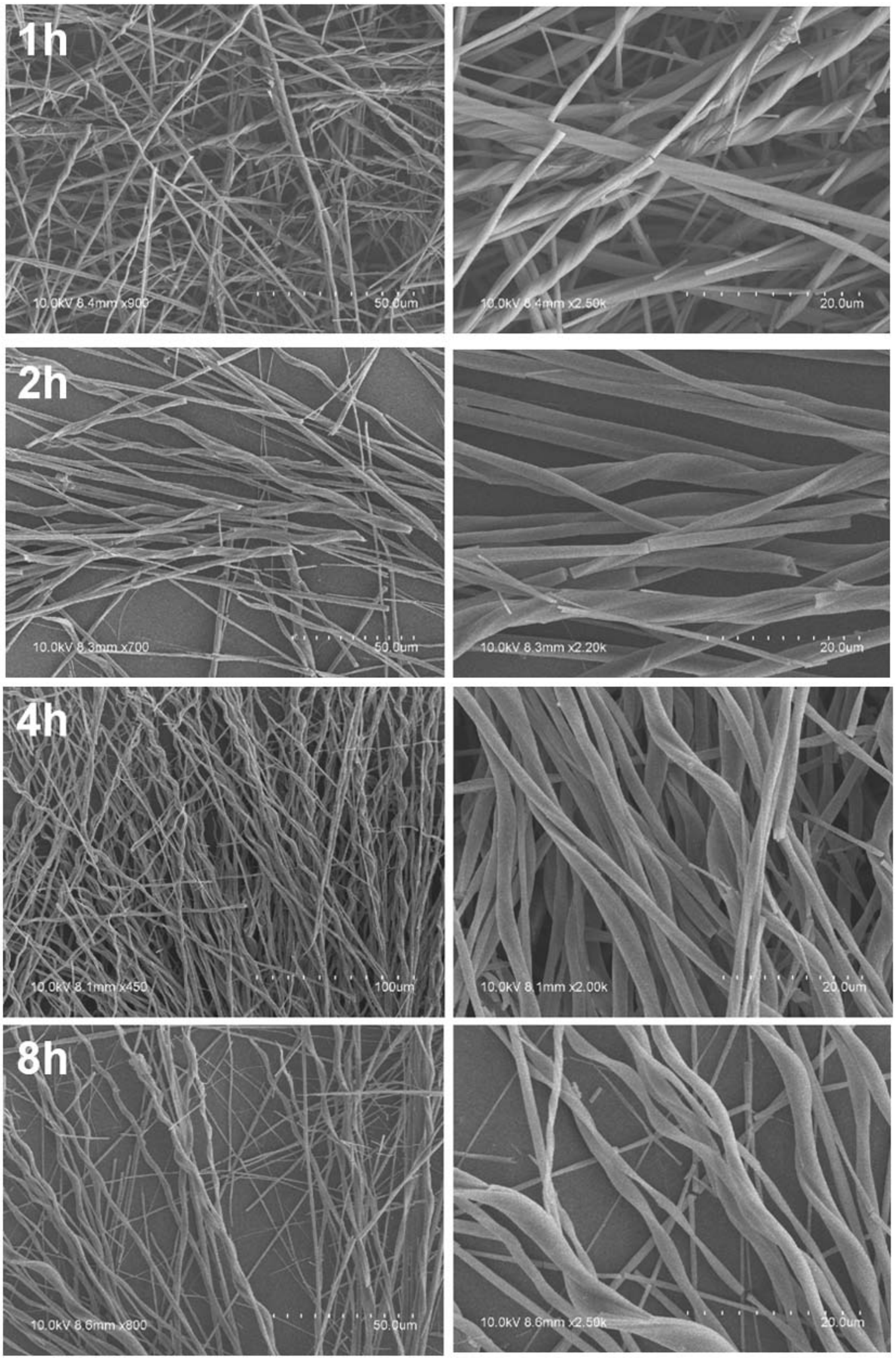
1h
10.0kV 8.4mm x900
50.0um
10.0kV 8.4mm x2.50k
20.0um
2h
10.0kV 8.3mm x700
50.0um
10.0kV 8.3mm x2.20k
20.0um
4h
10.0kV 8.1mm x450
100um
10.0kV 8.1mm x2.00k
20.0um
8h
10.0kV 8.6mm x800
50.0um
10.0kV 8.6mm x2.50k
20.0um

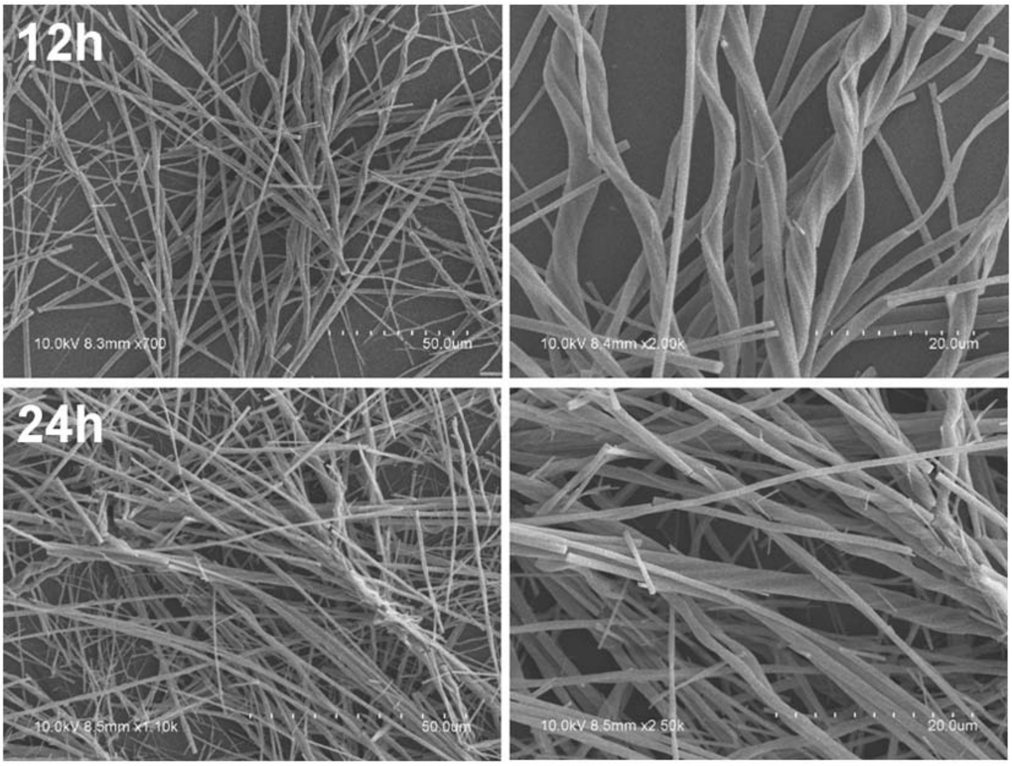

Supplementary Figure 12 | SEM images of the products after hydrothermal reactions of $Tb(NO_3)_3$ and *R*-$pempH_2$ at pH 3.1 and 120 °C for 1 - 24 h. The product obtained after 1 h had already taken the form of helical nanofibrils with different diameters and lengths.

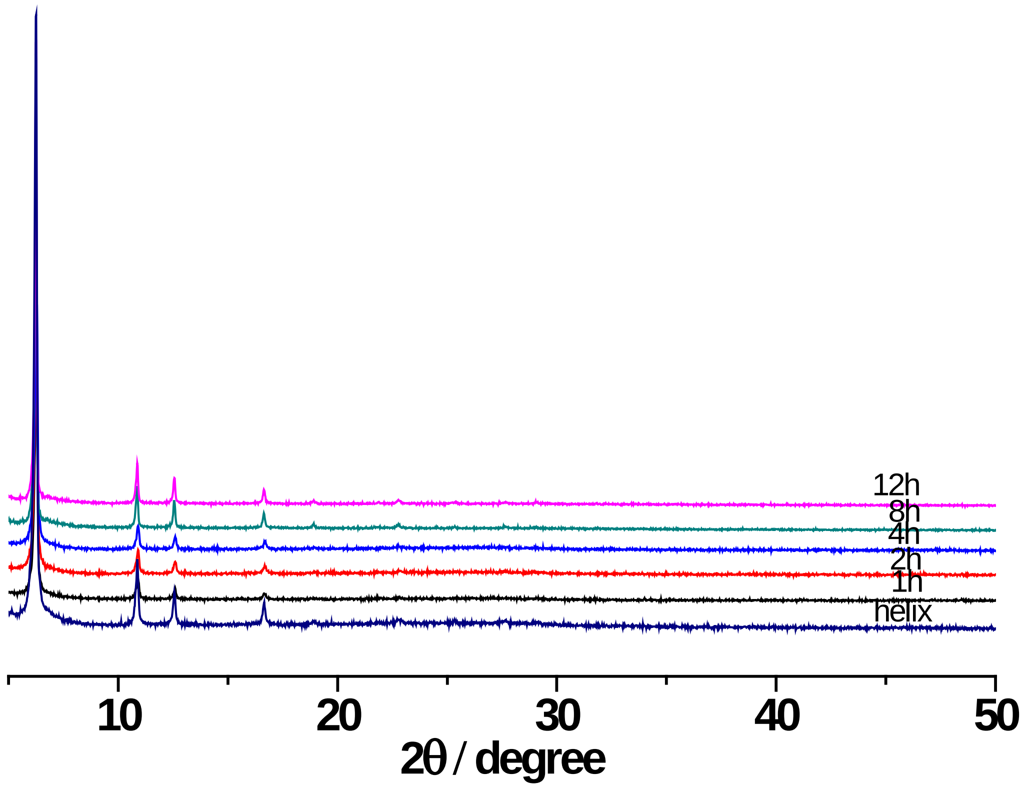

Supplementary Figure 13 | PXRD patterns of homochiral helices of ***R*-1** formed after hydrothermal reactions of $Tb(NO_3)_3$ and *R*-pempH$_2$ at pH 3.1 and 120 °C for 1 - 12 h.

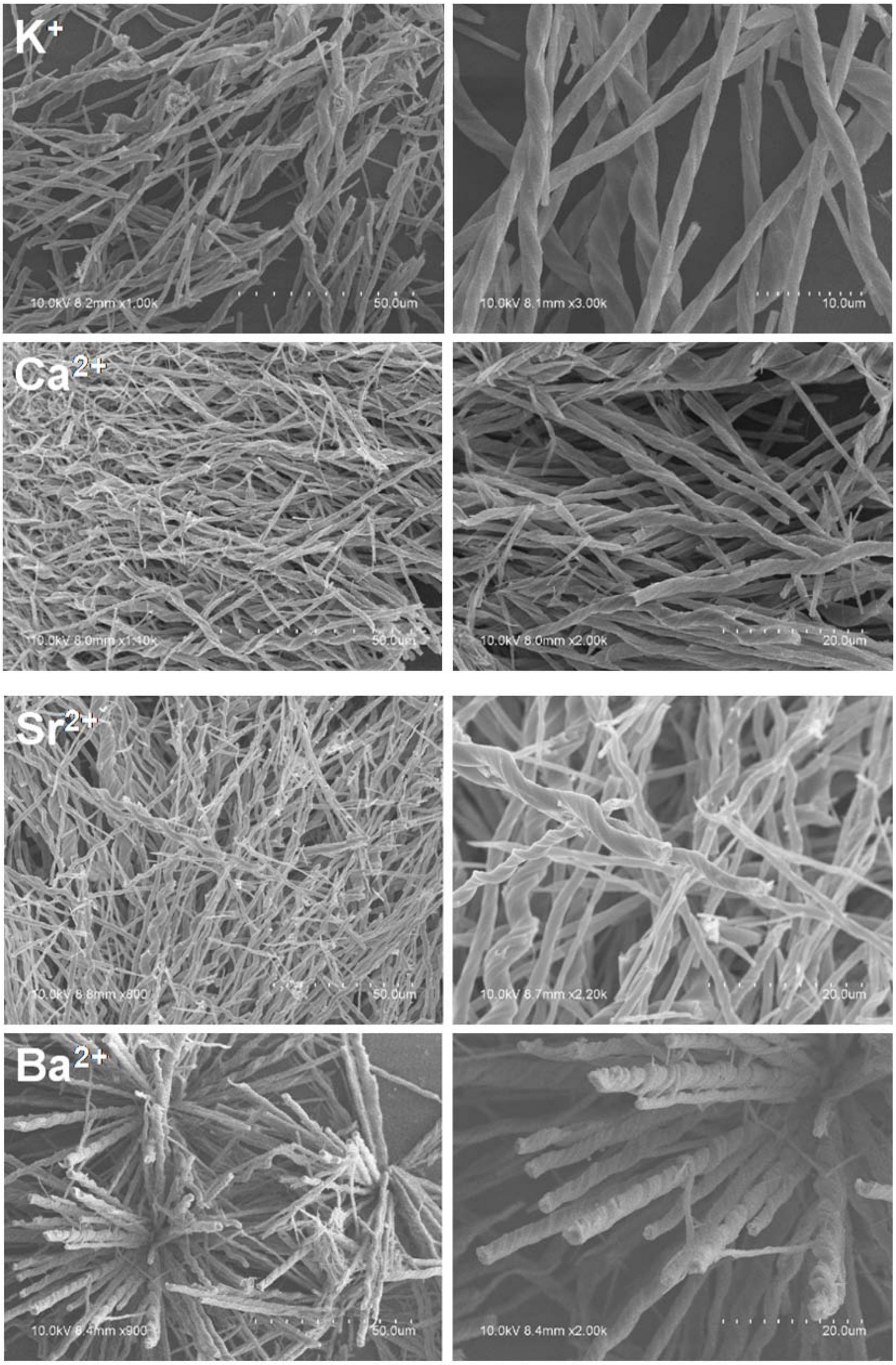

Supplementary Figure 14 | Homochiral helices of ***R*-1** formed after hydrothermal reactions of $Tb(NO_3)_3$ and *R*-pempH_2 at pH 3.1 and 120 °C for 2 d upon addition of different cations.

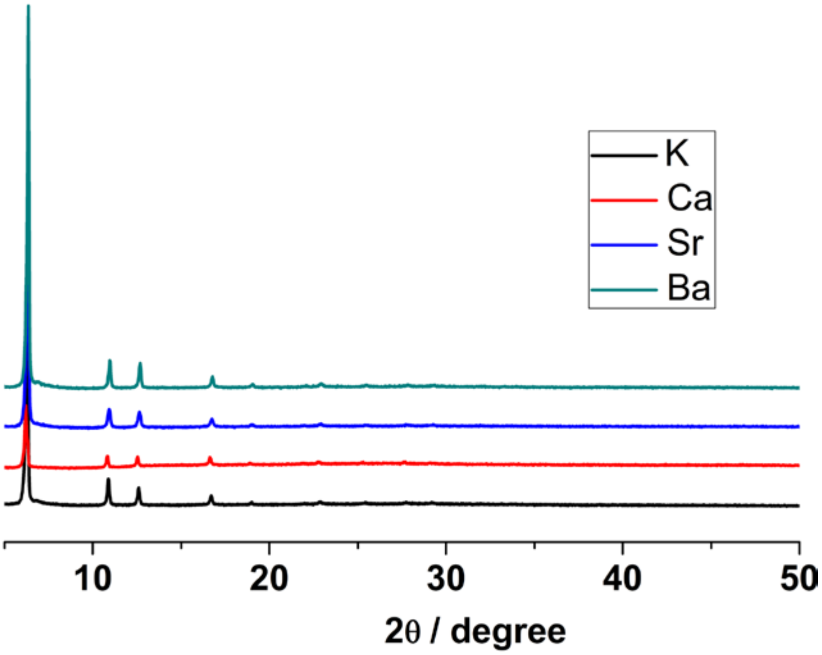

Supplementary Figure 15 | PXRD patterns of homochiral helices of ***R*-1** formed after hydrothermal reactions of $Tb(NO_3)_3$ and *R*-pempH_2 at pH 3.1 and 120 °C for 2 d in the presence of K^+, Ca^{2+}, Sr^{2+} and Ba^{2+}.

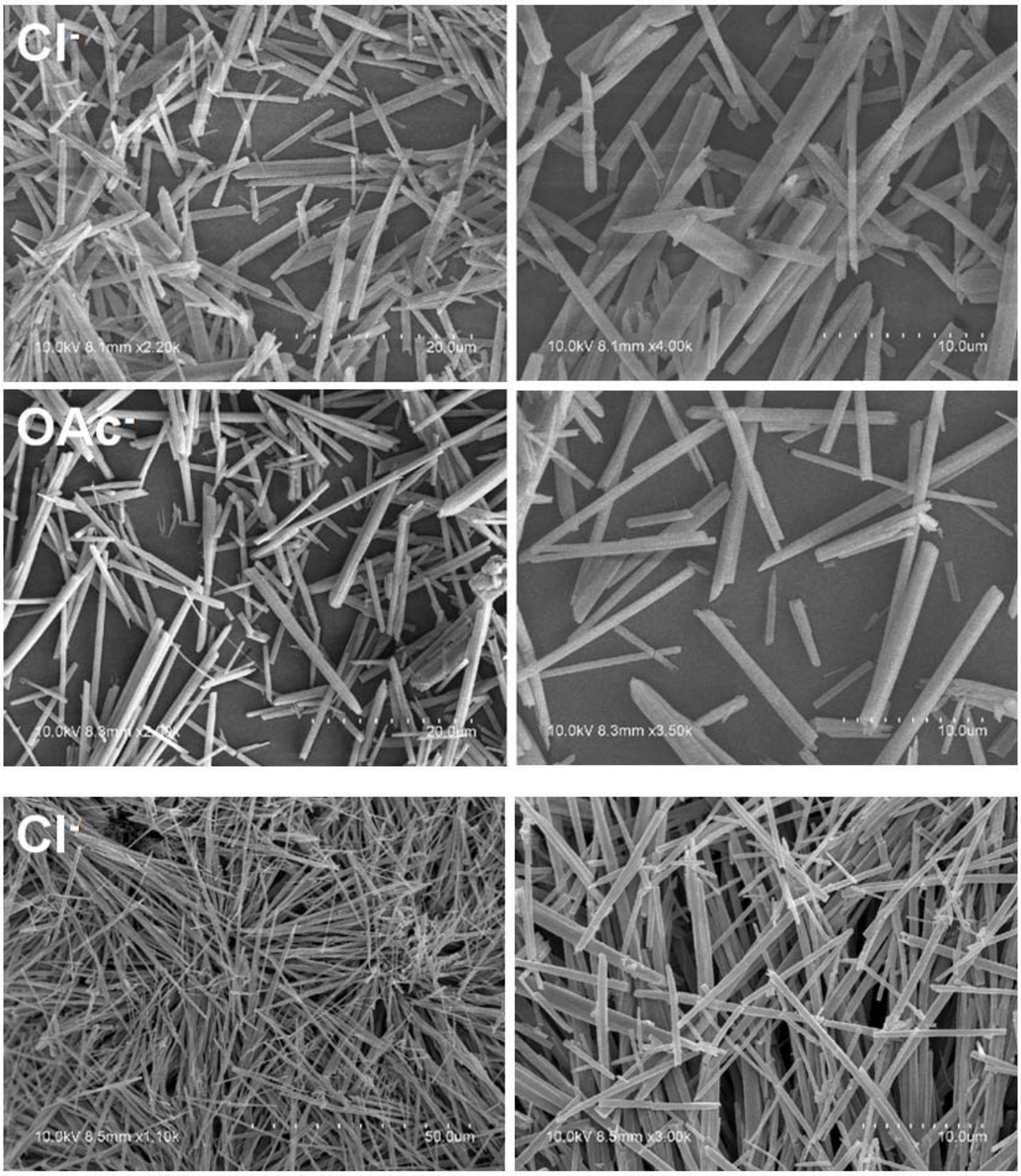

Supplementary Figure 16 | SEM images of products formed after hydrothermal reactions of TbX_3 (X = Cl, OAc, ClO_4) and *R*-pempH_2 at pH 3.1 and 120 °C for 2 d. No obvious helices were found in any of the reaction mixtures.

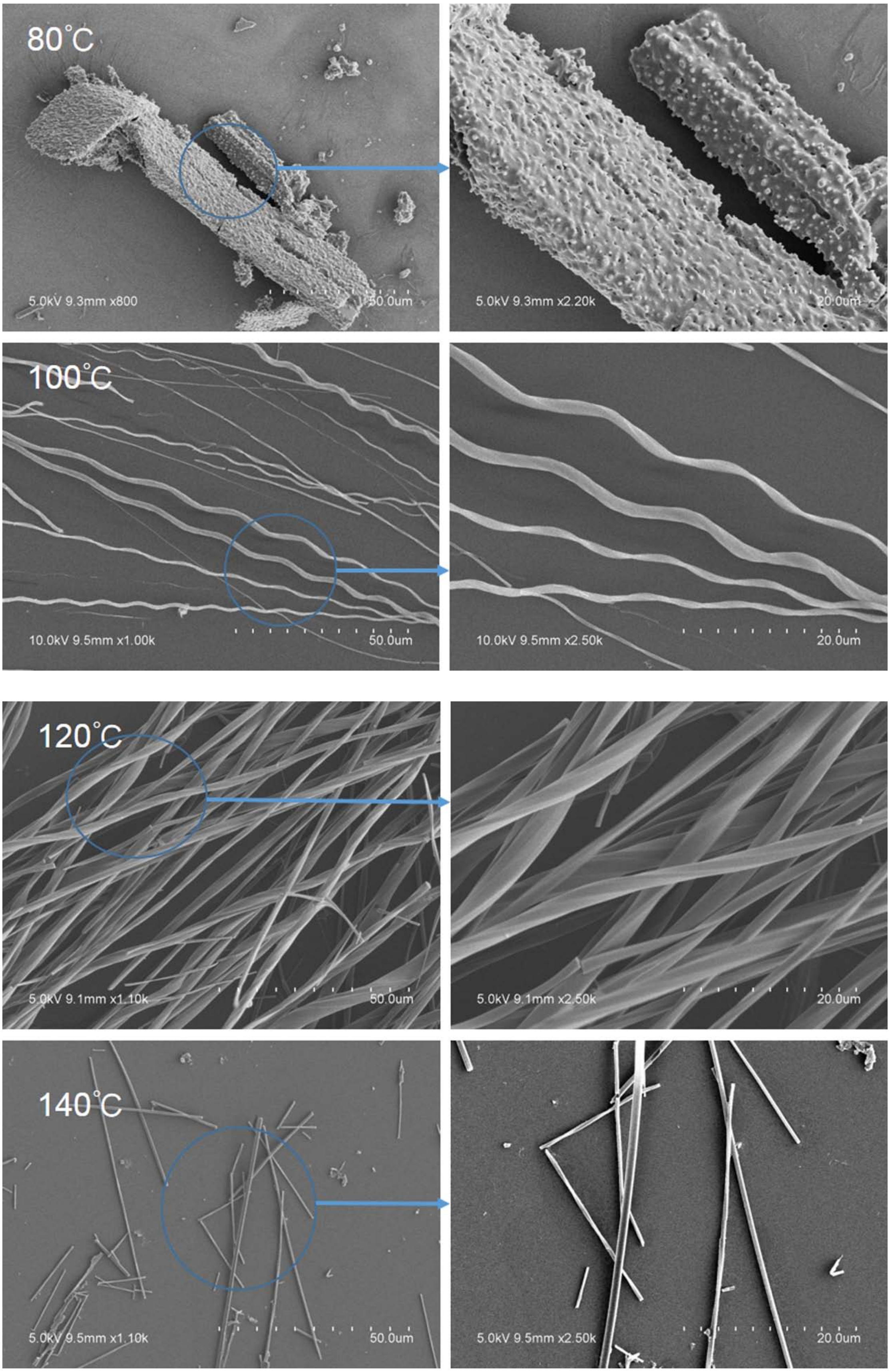
80˚C
5.0kV 9.3mm x800
50.0um
5.0kV 9.3mm x2.20k
20.0um
100˚C
10.0kV 9.5mm x1.00k
50.0um
10.0kV 9.5mm x2.50k
20.0um
120˚C
5.0kV 9.1mm x1.10k
50.0um
5.0kV 9.1mm x2.50k
20.0um
140˚C
5.0kV 9.5mm x1.10k
50.0um
5.0kV 9.5mm x2.50k
20.0um

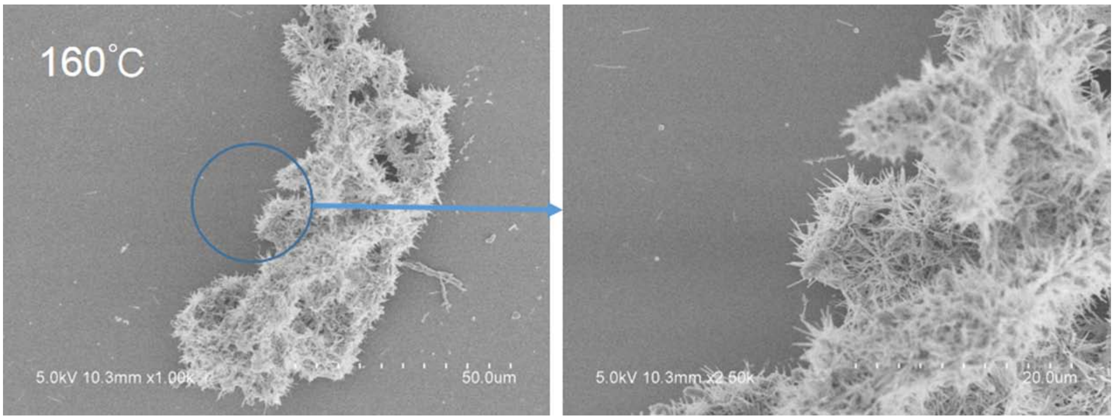

Supplementary Figure 17 | SEM images of products formed after hydrothermal reactions of $Tb(NO_3)_3$ and *R*-pempH_2 at pH 3.1 and 120 °C for 20 h. No obvious helices were found in the 80 ^oC and 160 ^oC products.

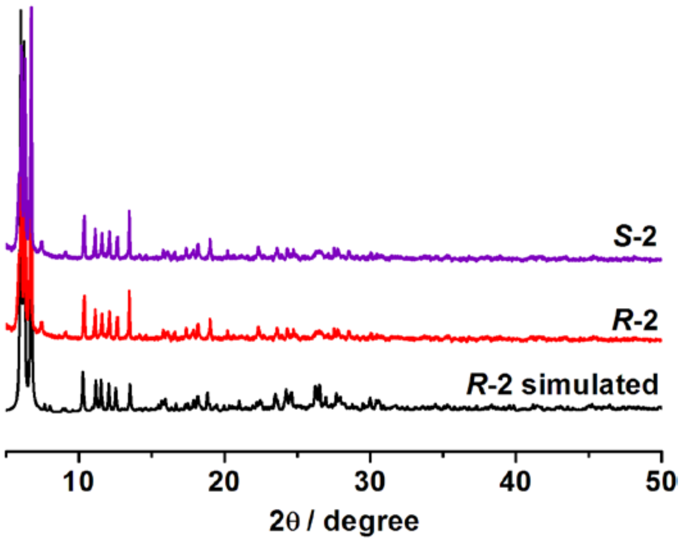

Supplementary Figure 18 | PXRD patterns of compounds ***R*-2** and ***S*-2**. The pattern simulated from the single crystal data for ***R*-2** is also given.

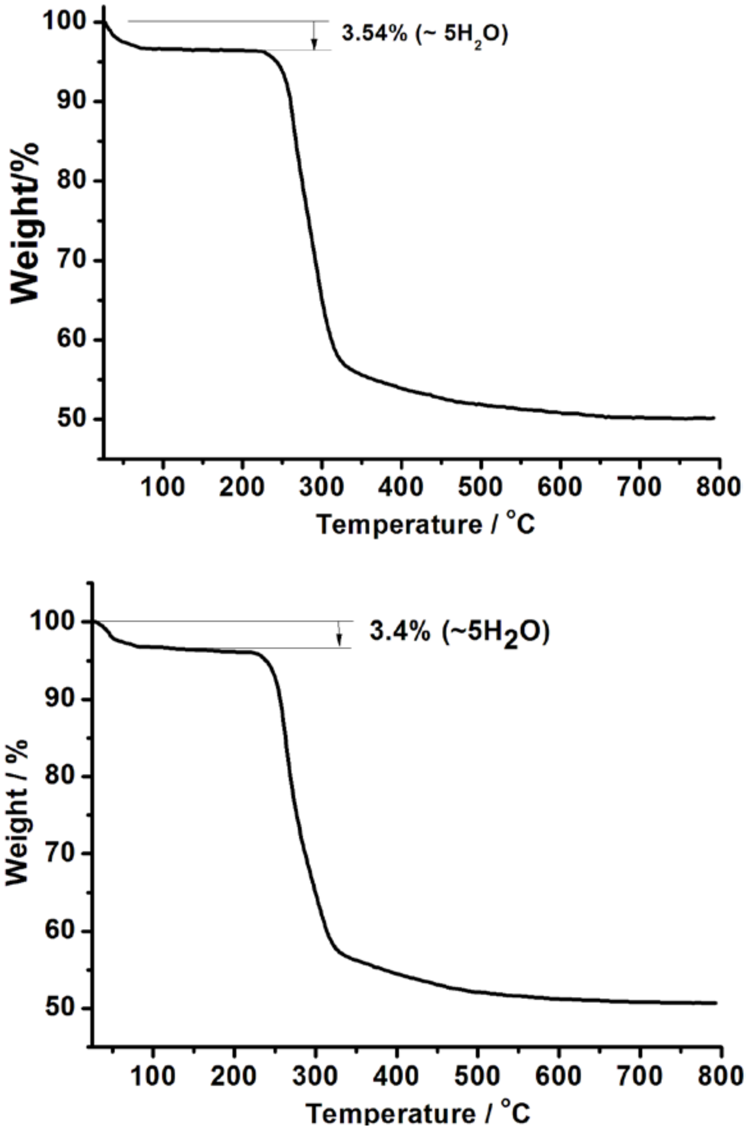

Supplementary Figure 19 | Thermal analysis curves of ***R*-2** (left) and ***S*-2** (right).

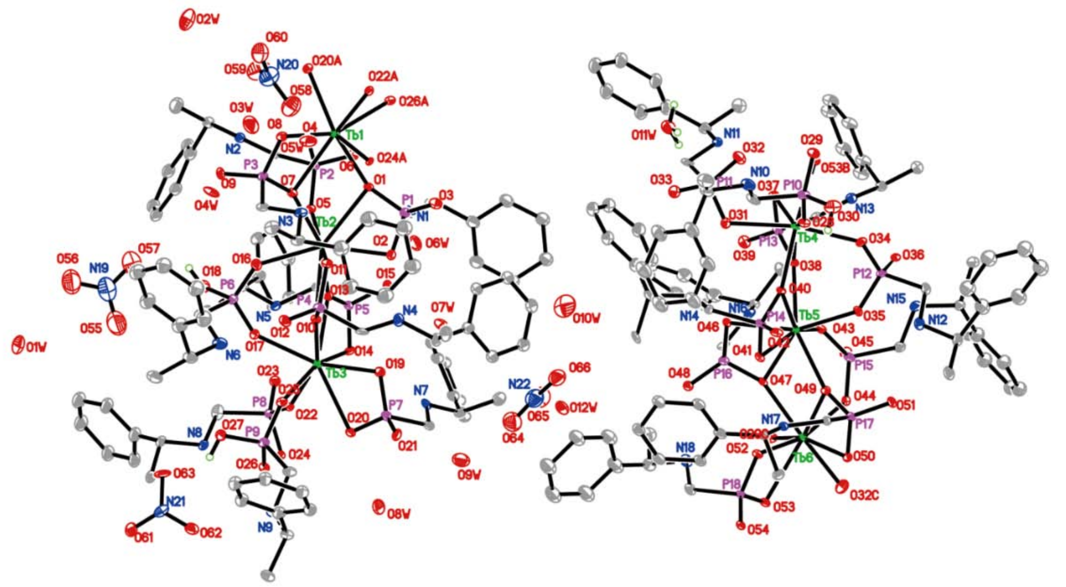

Supplementary Figure 20 | ORTEP view of ***R*-2** with 30% thermal ellipsoids. All H atoms are omitted for clarity except P-OH and H_3O^+. C atoms are not labeled for clarity.

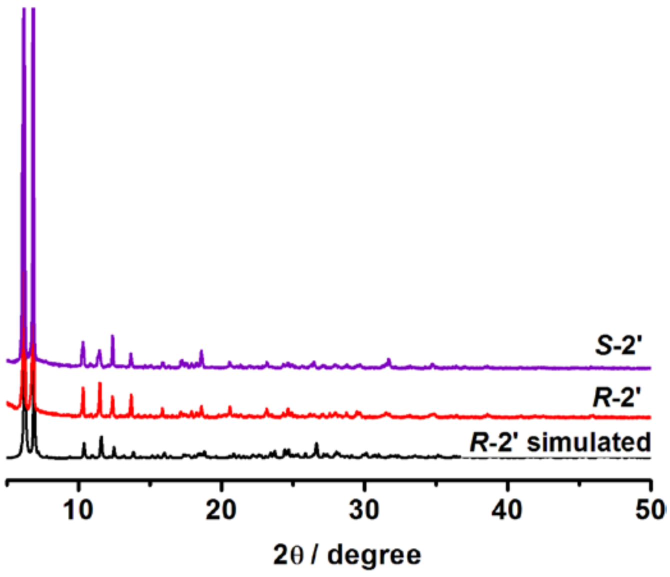

Supplementary Figure 21 | PXRD patterns of ***R*-2'** and ***S*-2'**. The pattern simulated from the single crystal data of ***R*-2'** is also shown.

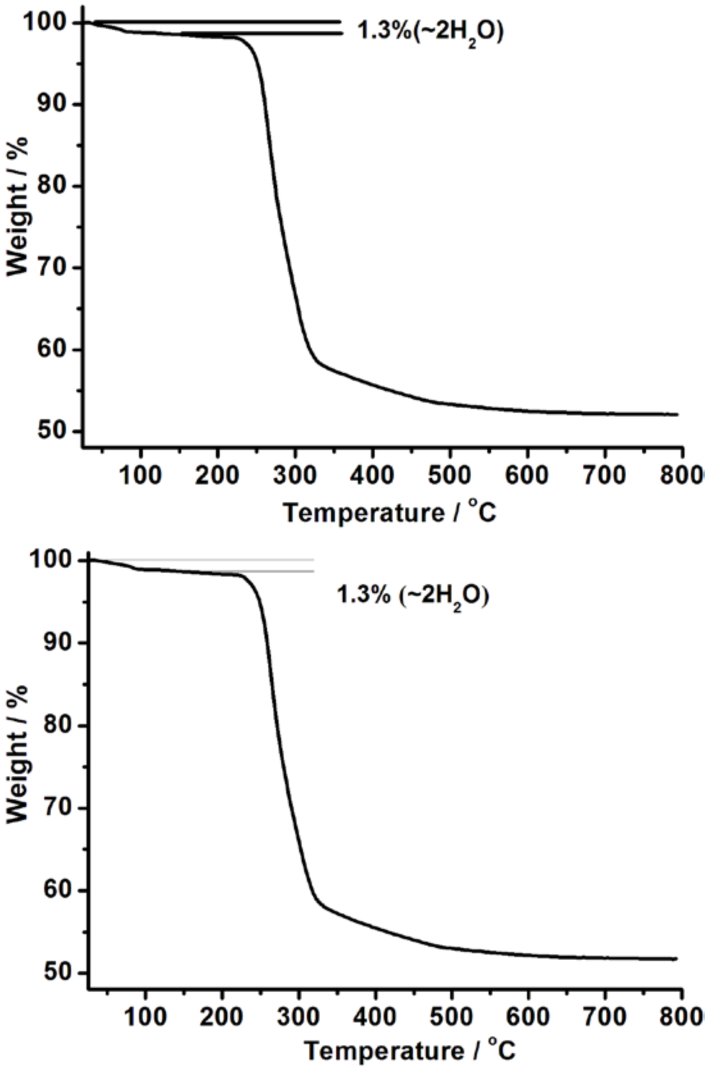

Supplementary Figure 22 | Thermal analysis curves of ***R*-2'** (left) and ***S*-2'** (right).

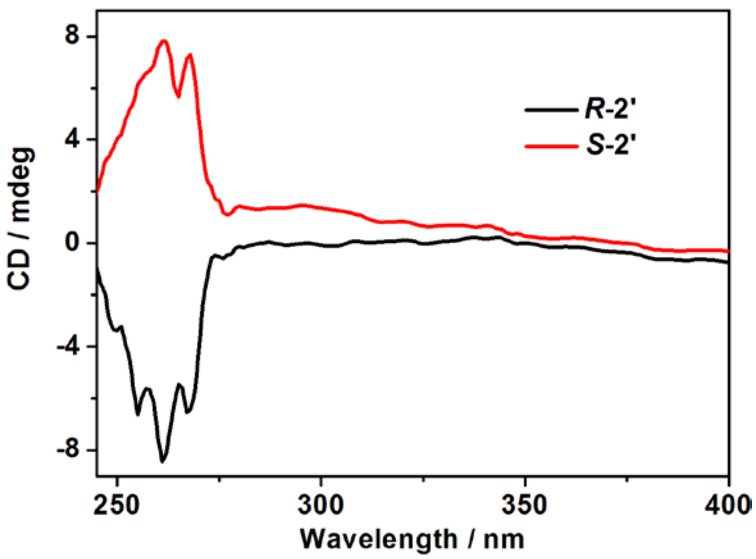

Supplementary Figure 23 | CD spectra of compounds ***R*-2'** and ***S*-2'**.

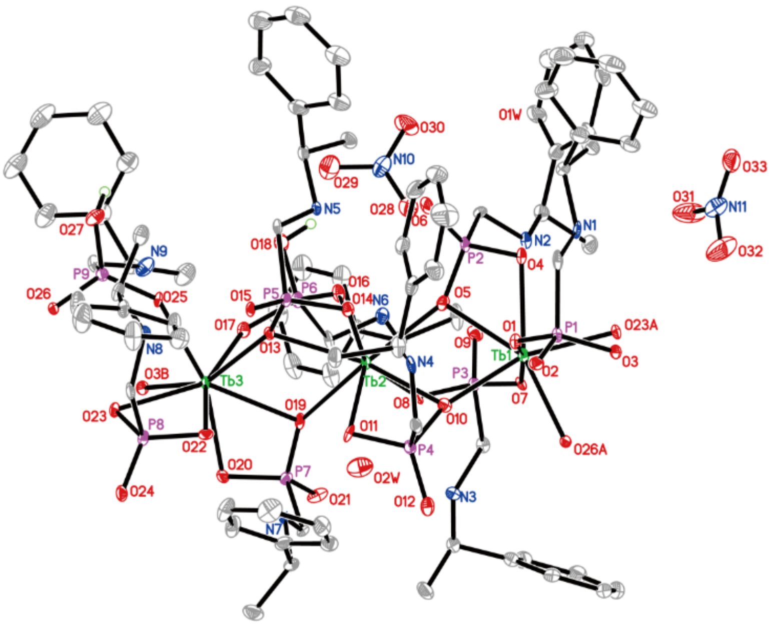

Supplementary Figure 24 | ORTEP view of ***R*-2'** with 30% thermal ellipsoids. All H atoms are omitted for clarity except P-OH. C atoms are not labelled for clarity.

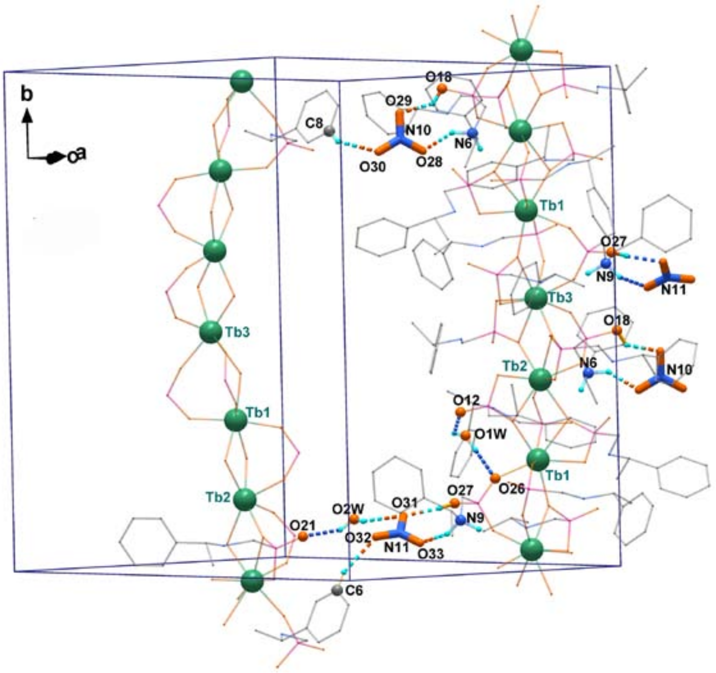

Supplementary Figure 25 | Hydrogen bonds in ***R*-2**' with NO_3^- anions and/or lattice water molecules.

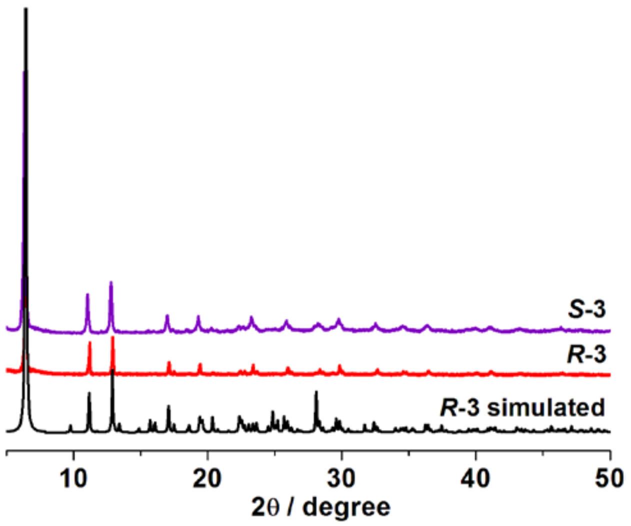

Supplementary Figure 26 | PXRD patterns of ***R*-3** and ***S*-3**. The pattern simulated from the single crystal data of ***R*-3** is also given.

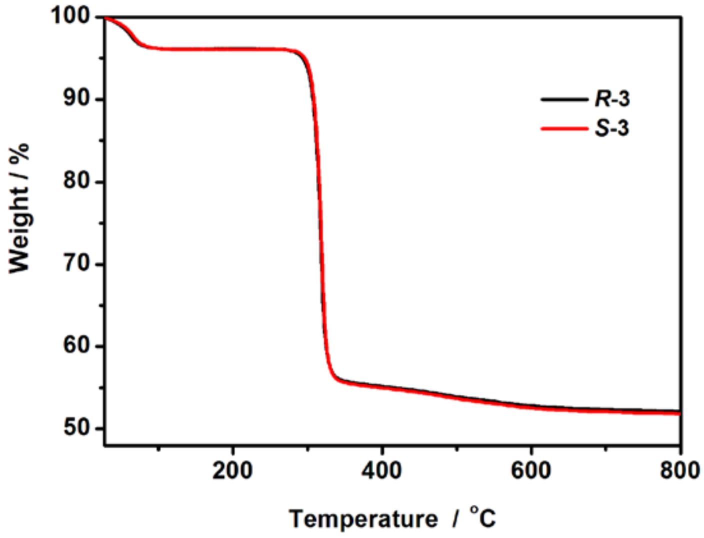

Supplementary Figure 27 | Thermal analysis curves of ***R*-3** and ***S*-3**.

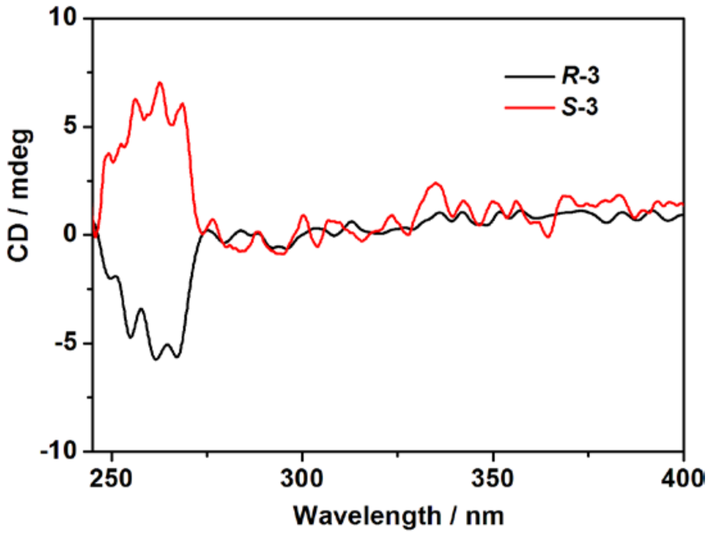

Supplementary Figure 28 | CD spectra of compounds ***R*-3** and ***S*-3**.

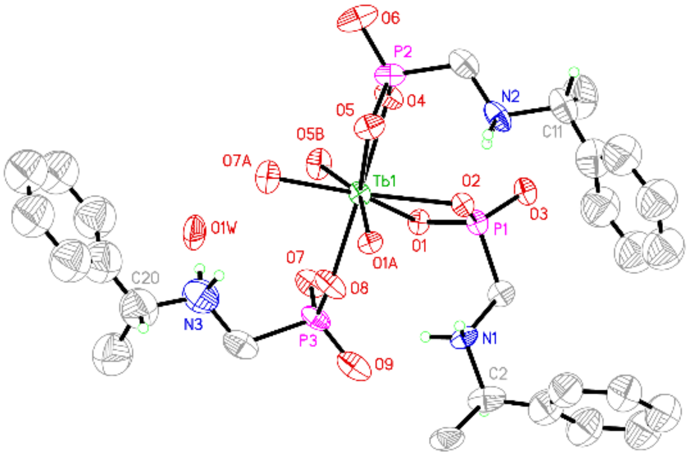

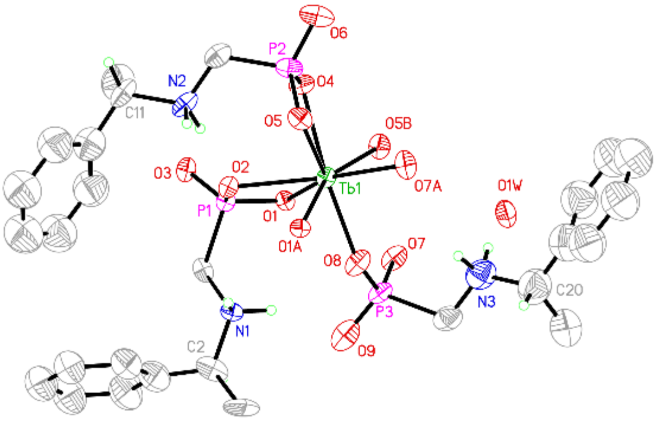

Supplementary Figure 29 | ORTEP view of ***R*-3** (top) and ***S*-3** (bottom) with 30% thermal ellipsoids. All C atoms except chiral C atoms are omitted for clarity.

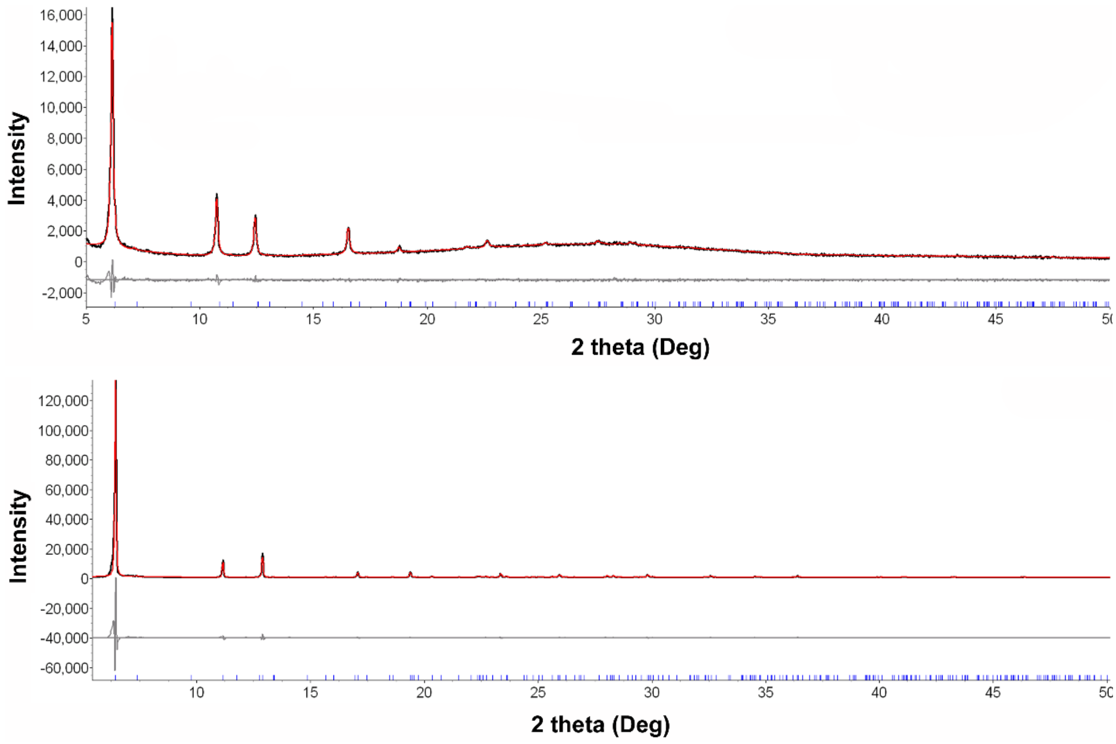

Supplementary Figure 30 | Pawley fit of powder samples of ***R*-1** (top) and ***R*-3** (bottom) using Topas 4.2 program.

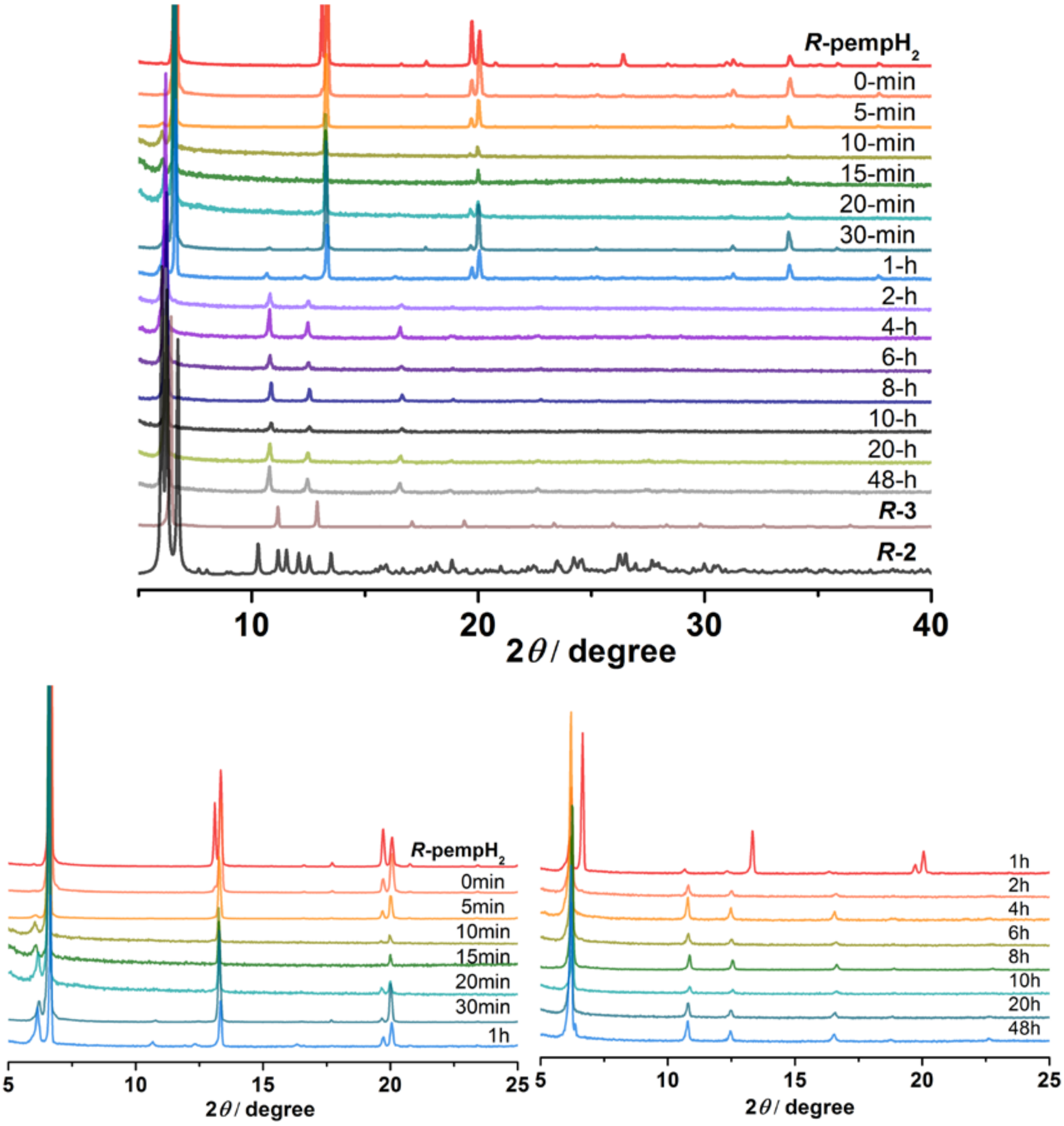

Supplementary Figure 31 | Top: The PXRD patterns of the products after hydrothermal reactions of $Tb(NO_3)_3$ and *R*-pempH_2 (pH ~ 3.1) at 120 °C for different period of time. The PXRD patterns of the ligand and the rod-like crystals of ***R*-3** as well as the block-like crystals of ***R*-2** are also given for a comparison. Bottom: Enlarged PXRD patterns of the same products for clarity.

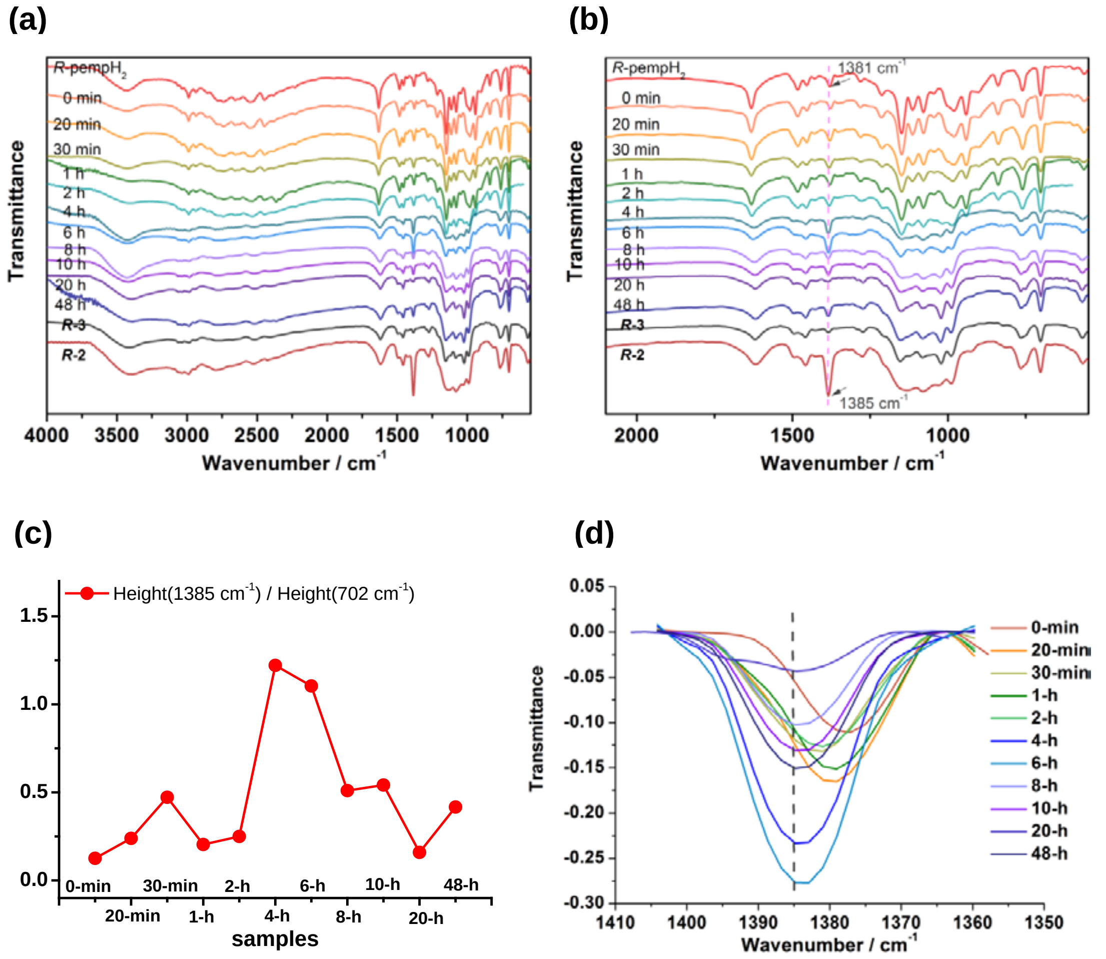

Supplementary Figure 32 | The IR spectra in the ranges of 540-4000 cm^{-1} (a) and 540-2100 cm^{-1} (b) of the products after hydrothermal reactions of $Tb(NO_3)_3$ and *R*-pempH$_2$ (pH ~ 3.1) at 120 °C for different period of time. The IR spectra of the ligand, the rod-like crystals of ***R*-3** and the block-like crystals of ***R*-2** are also given for a comparison. (c) The variation of the peak intensity at 1385 cm^{-1} for the time-depentent products. The peak at 702 cm^{-1} was assigned to the vibration of mono-substituted phenyl, which was used as the internal standard to eliminate the differences caused by the concentrations of different samples. (d) The normalized spectra with the baseline subtracted, from which the peak intensity at 1385 cm^{-1} was abstracted.

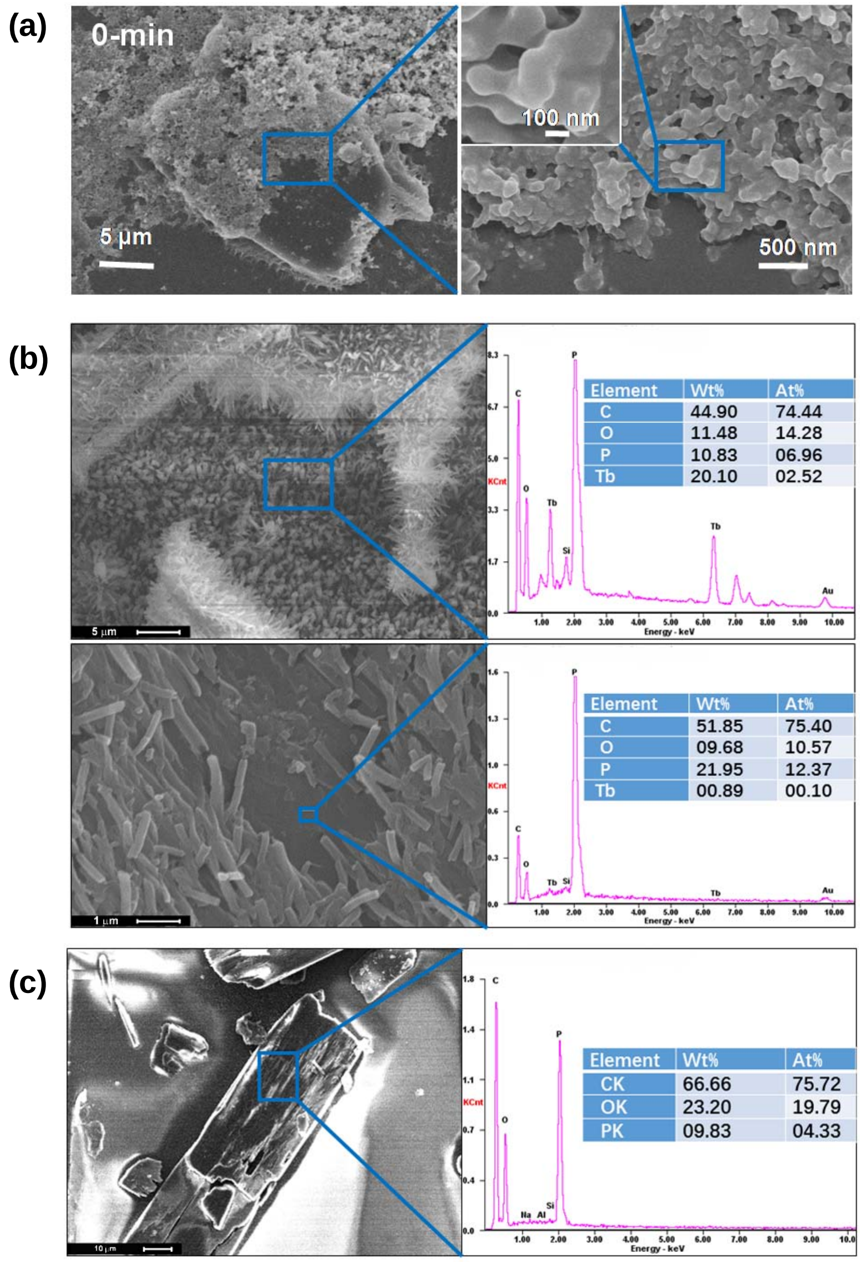

Supplementary Figure 33 | (a) SEM images of 0-min product. (b) EDX analysis of the 0-min product in different regions. (c) EDX analysis of *R*-pempH_2, after stirring in 6 mL water for 2 hours.

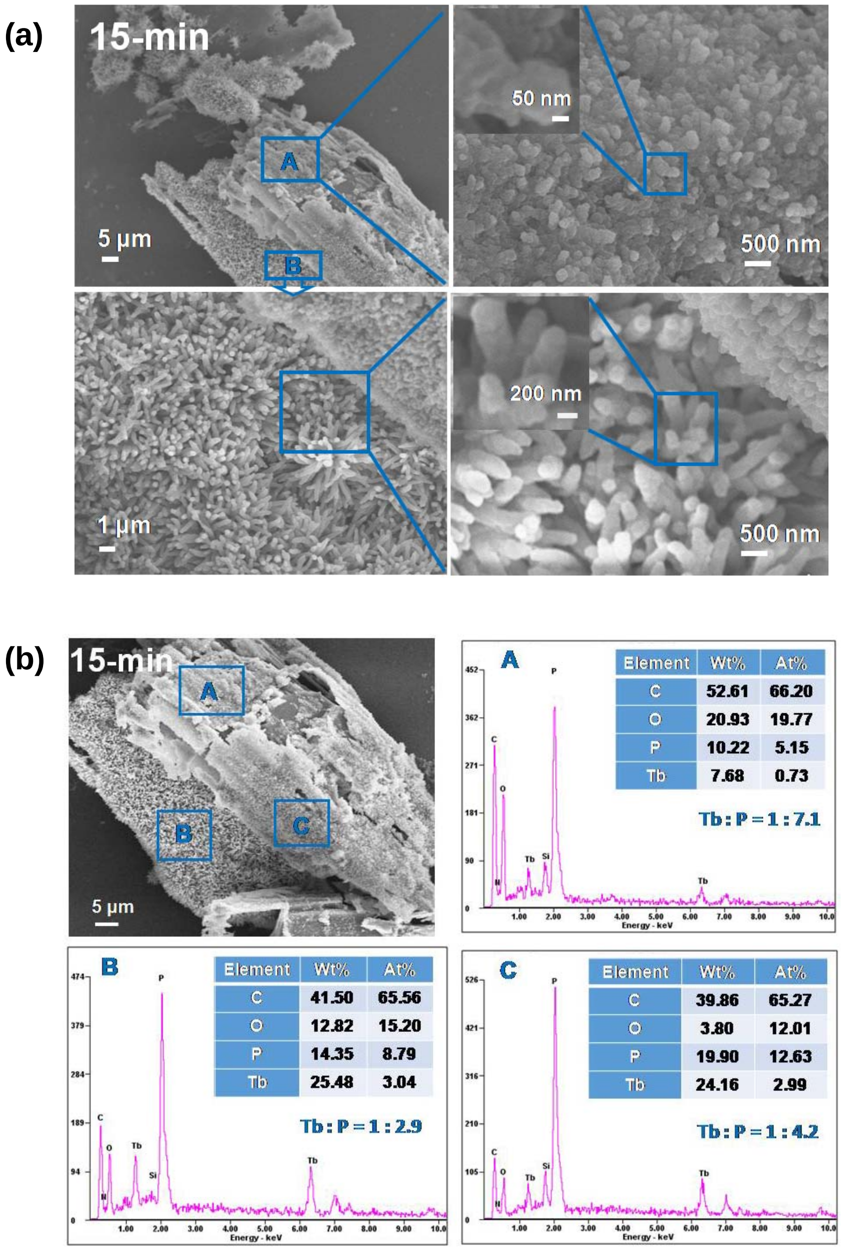

Supplementary Figure 34 | (a) SEM images of the 15-min product. (b) EDX analyses of the 15-min product in different regions.

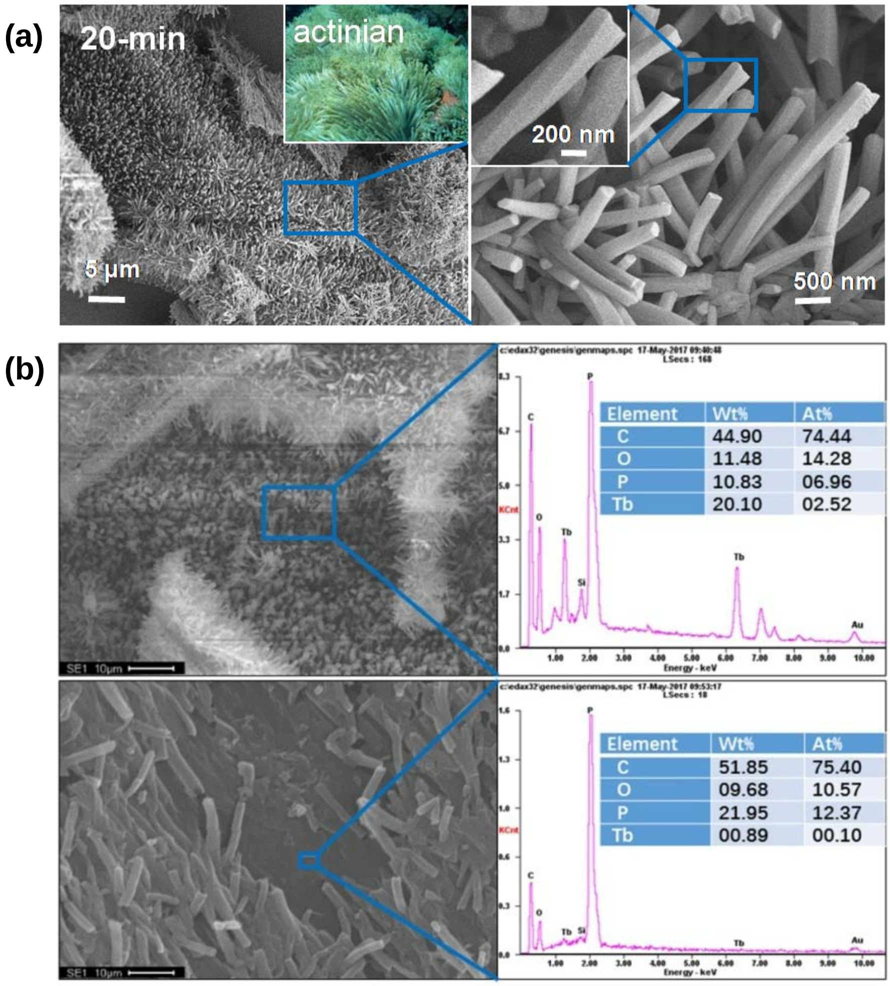

Supplementary Figure 35 | SEM images (a) and EDX analyses (b) of the 20-min product.

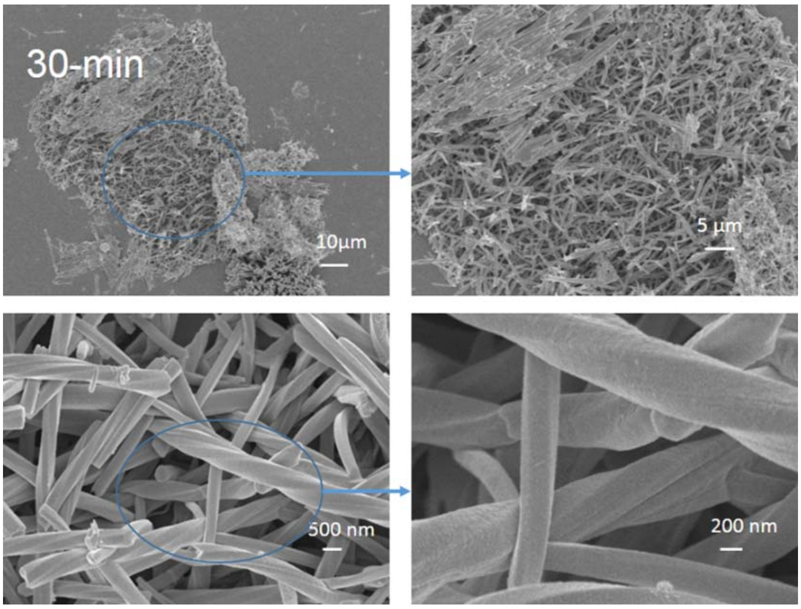

Supplementary Figure 36 | SEM images of the 30-min products.

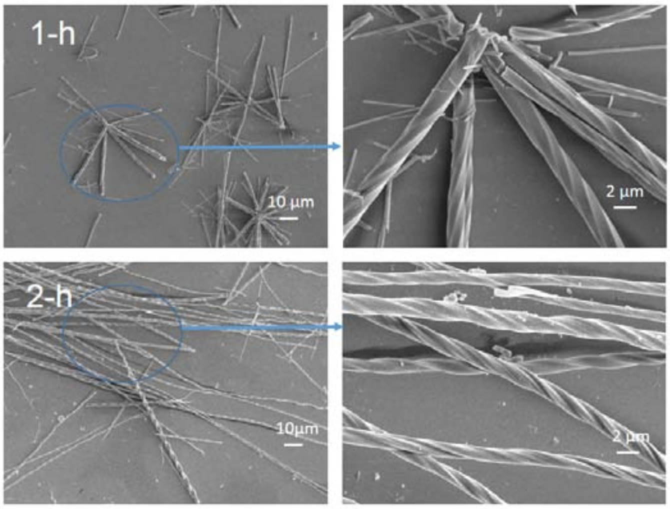

Supplementary Figure 37 | SEM images of the 1-h and 2-h products.

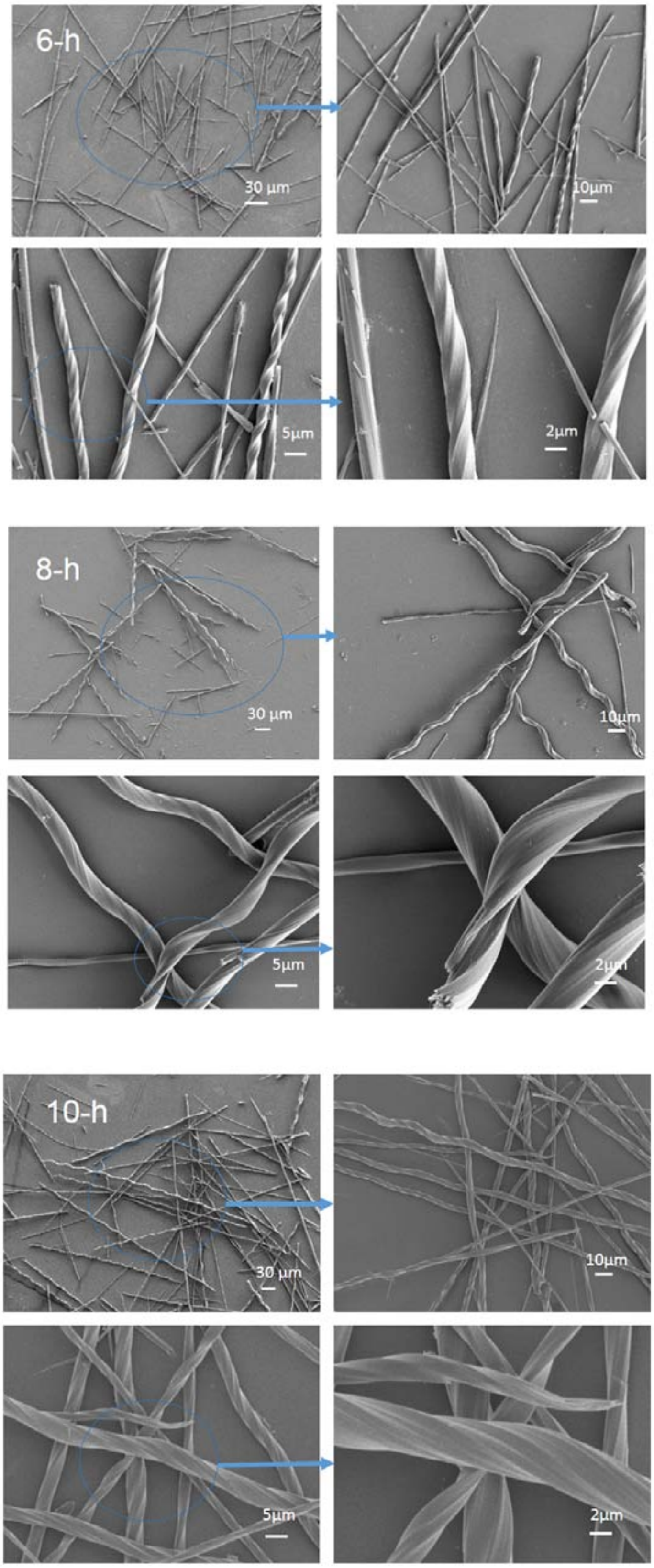
6-h
30 µm
10µm
5µm
2µm
8-h
30 µm
10µm
5µm
2µm
10-h
30 µm
10µm
5µm
2µm

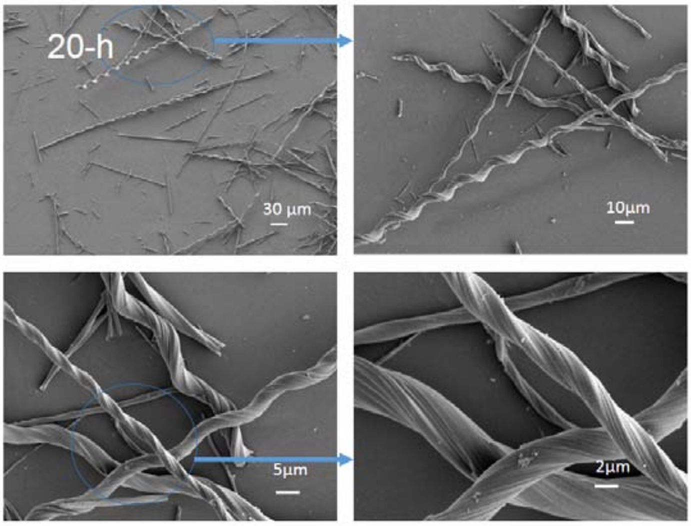

Supplementary Figure 38 | SEM images of the 6-h, 8-h, 10-h and 20-h products.

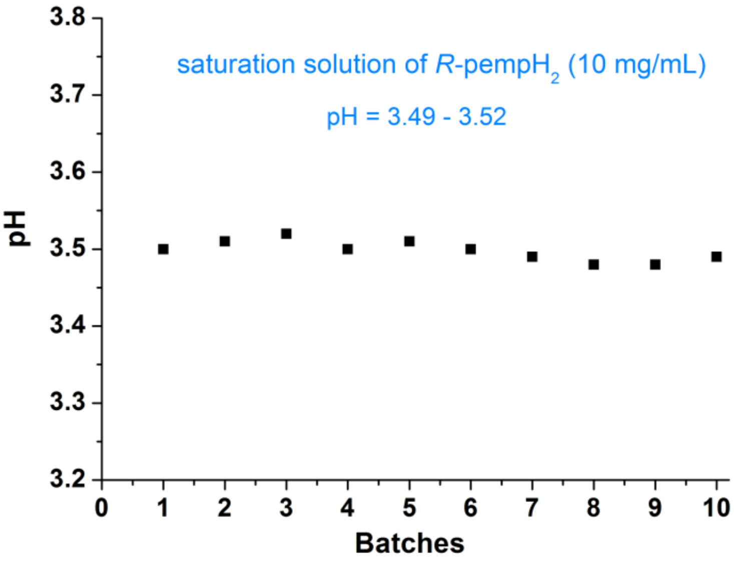

Supplementary Figure 39 | The pH values of the saturated solutions of *R*-pempH_2 (10 mg/mL) measured for ten parallel batches.

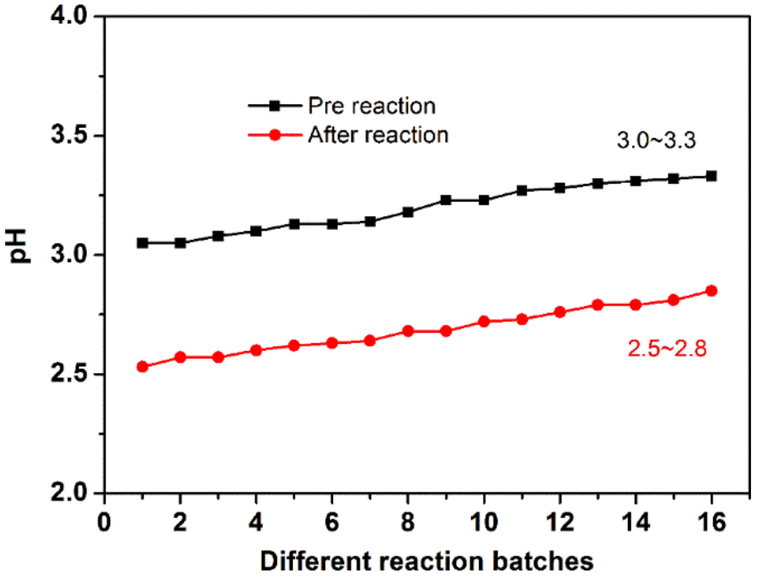

Supplementary Figure 40 | The pH value of the systems measured before and after hydrothermal reactions of $Tb(NO_3)_3$ and *R*-pempH$_2$ at 120 °C for 2 d.

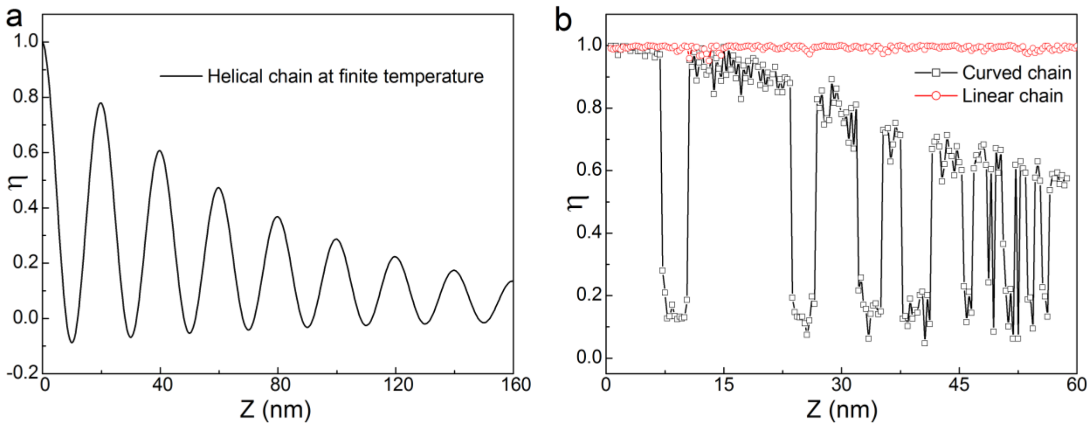

Supplementary Figure 41 | Tangent correlation functions (η) for different types of chains. η is defined as[1]: $\eta = \boldsymbol{t_z} \cdot \boldsymbol{t_0}$, where $\boldsymbol{t_z}$ is the mean tangent vector at the coordinate z. (a) Theoretical results of tangent correlation function for helical chain at finite temperature[1]. (b) Simulation results of tangent correlation functions for linear chain (red line) in left panel of Figure 9a and curved chain (black line) in right panel of Figure 9c.

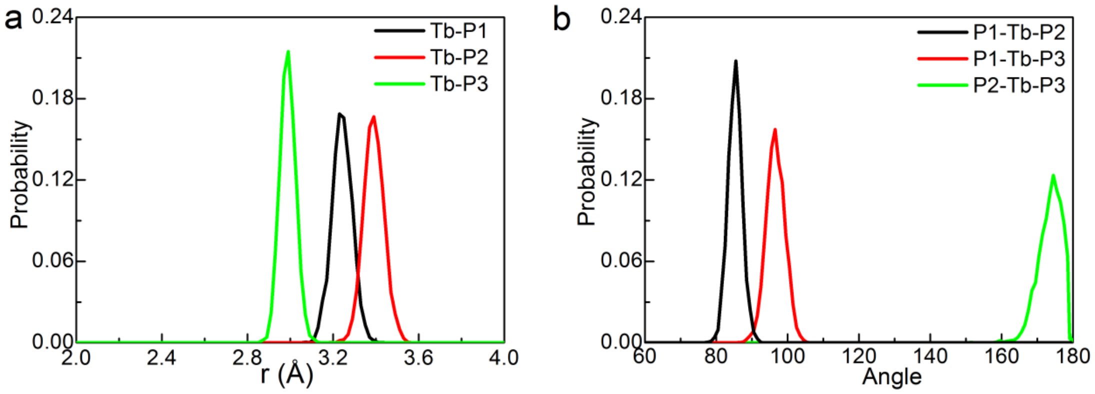

Supplementary Figure 42 | The cumulative probability distribution of Tb-P distance (a) and P-Tb-P angle (b) in Tb^{3+}-*R*-pempH^{-} system in all-atom simulation (averaged over 100 ns). The peak in (a) is 2.99 Å, 3.23 Å, 3.39 Å, respectively; while the Tb-P distance in the experiments is 3.07 Å, 3.13 Å, 3.55 Å, respectively. The peak value in (b) is 85.50°, 96.50°, 174.50°, respectively; while the P-Tb-P angle in the experiments is 83.00°, 98.66°, 173.35°, respectively.

Supplementary Table 1 | Energy dispersive X-ray spectroscopy (EDX) analyses of ***R*-1** and ***S*-1** helices.

	***R*-1**		***S*-1**	
Element	***Wt%***	***At%***	***Wt%***	***At%***
CK	56.60	77.36	46.48	69.52
NK	02.21	02.58	05.22	06.70
OK	14.25	14.62	14.87	16.69
PK	06.68	03.54	07.75	04.49
TbL	10.63	01.10	11.93	01.35
AuL	09.64	00.80	13.75	01.25
total	100.00	100.00	100.00	100.00

Supplementary Table 2 | Elemental analyses of ***R*-1**, ***S*-1**, ***R*-3** and ***S*-3**.

	Calculated	***R*-3**	***R*-1**	***S*-3**	***S*-1**
H/%	5.17	5.24	5.25	5.24	5.27
C/%	38.72	38.92	38.67	38.99	38.73
N/%	5.02	5.09	4.98	4.93	4.91

Supplementary Table 3 | Crystal data and structure refinements for ***R*-2**, ***R*-2'**, ***R*-3** and ***S*-3**.

**Compound**	***R*-2**	***R*-2'**	***R*-3**	***S*-3**	***R*-3**	***S*-3**
Formula	$C_{162}H_{262}N_{22}O_{78}P_{18}Tb_6$	$C_{81}H_{123}N_{11}O_{35}P_9Tb_3$	$(C_{27}H_{39}N_3O_9P_3Tb)_2(H_2O)$	$(C_{27}H_{39}N_3O_9P_3Tb)_2(H_2O)$	$(C_{27}H_{39}N_3O_9P_3Tb)_2(H_2O)$	$(C_{27}H_{39}N_3O_9P_3Tb)_2(H_2O)$
M	5276.90	2566.39	1620.90	1620.90	1620.90	1620.90
crystal size [mm]	0.25 ×0.25× 0.20	0.30× 0.20× 0.15	0.40 × 0.10× 0.10	0.30×0.05× 0.05	0.30 × 0.10× 0.10	0.60 × 0.15× 0.15
crystal system	Monoclinic	Orthorhombic	Hexagonal	Hexagonal	Hexagonal	Hexagonal
space group	$P2_1$	$P2_12_12_1$	$P6_5$	$P6_1$	$P6_5$	$P6_1$
T (K)	150	150	150	150	277	277
a [Å]	17.196(3)	16.987(2)	15.8238(4)	15.7762(4)	15.9914(4)	15.9695(3)
b [Å]	24.279(4)	24.174(3)	15.8238(4)	15.7762(4)	15.9914(4)	15.9695(3)
c [Å]	26.223(5)	25.552(3)	24.1596(14)	24.1029(13)	24.2935(14)	24.2768(9)
α [°]	90	90	90	90	90	90
β [°]	92.282(4)	90	90	90	90	90
γ [°]	90	90	120	120	120	120
V [Å^3], Z	10940(3), 2	10493(2), 4	5238.9(5), 3	5195.2(4), 3	5380.1(5), 3	5361.7(3), 3
D_c [g cm^{-3}]	1.602	1.625	1.541	1.554	1.501	1.506
μ[mm^{-1}]	2.315	2.221	2.214	2.233	2.156	2.163
F(000), R_{int}	5344, 0.153	5184, 0.079	2454, 0.067	2454, 0.065	2454, 0.071	2454, 0.040
T_{max},T_{min}, GoF on F^2	0.660, 0.590, 1.02	0.720, 0.590, 1.13	0.871, 0.500, 1.00	0.897, 0.554, 1.00	0.810, 0.564, 1.03	0.738, 0.650, 1.01
R_1, wR_2 [I>2σ(I)]	0.0992, 0.1815	0.0642, 0.1561	0.0762, 0.1954	0.0723, 0.1905	0.0821, 0.2065	0.0755, 0.1977
(all data)	0.1690, 0.2075	0.0776, 0.1613	0.0988, 0.2165	0.0905, 0.2077	0.1185, 0.2461	0.0936, 0.2223
Flack parameter	0.027(13)	0.026(4)	0.007(11)	0.014(9)	0.02(5)	0.011(9)
$(\Delta\rho)_{max}$,$(\Delta\rho)_{min}$ / e Å^{-3}	4.98, -3.15	3.36, -3.27	5.46, -1.57	5.78, -1.91	4.24, -2.61	4.74, -2.66
CCDC number	1501001	1501002	1501003	1501004	1501005	1501006

Supplementary Table 4 | Selected bond lengths [Å] and angles [˚] for ***R*-2**.

Tb(1)-O(1)	2.358(12)	Tb(3)-O(13)	2.475(12)	P(4)-O(11)	1.491(13)
Tb(1)-O(4)	2.285(11)	Tb(3)-O(14)	2.359(12)	P(4)-O(12)	1.551(13)
Tb(1)-O(7)	2.504(12)	Tb(3)-O(17)	2.394(13)	P(5)-O(13)	1.557(13)
Tb(1)-O(8)	2.382(12)	Tb(3)-O(19)	2.370(13)	P(5)-O(14)	1.515(13)
Tb(1)-O(20)#1	2.413(12)	Tb(3)-O(20)	2.619(12)	P(5)-O(15)	1.557(13)
Tb(1)-O(22)#1	2.532(12)	Tb(3)-O(22)	2.358(12)	P(6)-O(16)	1.494(13)
Tb(1)-O(24)#1	2.417(11)	Tb(3)-O(25)	2.374(12)	P(6)-O(17)	1.514(13)
Tb(1)-O(26)#1	2.467(12)	P(1)-O(1)	1.526(12)	P(6)-O(18)	1.553(14)
Tb(2)-O(1)	2.583(12)	P(1)-O(2)	1.517(14)	P(7)-O(19)	1.490(12)
Tb(2)-O(2)	2.396(11)	P(1)-O(3)	1.520(13)	P(7)-O(20)	1.528(13)
Tb(2)-O(5)	2.277(10)	P(2)-O(4)	1.545(13)	P(7)-O(21)	1.536(12)
Tb(2)-O(7)	2.390(12)	P(2)-O(5)	1.551(11)	P(8)-O(22)	1.519(12)
Tb(2)-O(10)	2.650(11)	P(2)-O(6)	1.542(13)	P(8)-O(23)	1.507(13)
Tb(2)-O(11)	2.415(11)	P(3)-O(7)	1.514(14)	P(8)-O(24)	1.529(13)
Tb(2)-O(13)	2.362(12)	P(3)-O(8)	1.576(11)	P(9)-O(25)	1.529(13)
Tb(2)-O(16)	2.347(13)	P(3)-O(9)	1.538(13)	P(9)-O(26)	1.515(13)
Tb(3)-O(10)	2.327(10)	P(4)-O(10)	1.552(13)	P(9)-O(27)	1.583(13)
Tb(4)-O(53)#2	2.326(12)	Tb(6)-O(49)	2.448(10)	P(13)-O(39)	1.483(14)
Tb(4)-O(34)	2.322(11)	Tb(6)-O(50)	2.468(11)	P(14)-O(40)	1.551(11)
Tb(4)-O(28)	2.313(13)	Tb(6)-O(29)#3	2.342(13)	P(14)-O(41)	1.536(11)
Tb(4)-O(31)	2.416(13)	Tb(6)-O(32)#3	2.339(13)	P(14)-O(42)	1.456(14)
Tb(4)-O(40)	2.306(10)	Tb(6)-O52)	2.375(10)	P(15)-O(43)	1.553(13)
Tb(4)-O(37)	2.444(13)	Tb(6)-O(53)	2.581(12)	P(15)-O(44)	1.555(13)
Tb(4)-O(38)	2.449(12)	P(10)-O(28)	1.511(13)	P(15)-O(45)	1.499(14)
Tb(5)-O(43)	2.357(12)	P(10)-O(29)	1.513(13)	P(16)-O(46)	1.520(13)
Tb(5)-O(41)	2.406(12)	P(10)-O(30)	1.571(14)	P(16)-O(47)	1.538(11)
Tb(5)-O(46)	2.473(11)	P(11)-O(31)	1.521(13)	P(16)-O(48)	1.547(11)
Tb(5)-O(47)	2.448(10)	P(11)-O(32)	1.507(13)	P(17)-O(49)	1.498(11)
Tb(5)-O(49)	2.351(10)	P(11)-O(33)	1.503(13)	P(17)-O(50)	1.565(13)
Tb(5)-O(40)	2.542(10)	P(12)-O34)	1.541(13)	P(17)-O(51)	1.515(14)
Tb(5)-O(35)	2.287(11)	P(12)-O(35)	1.528(13)	P(18)-O(52)	1.576(13)
Tb(5)-O(38)	2.366(12)	P(12)-O(36)	1.531(13)	P(18)-O(53)	1.588(13)
Tb(6)-O(44)	2.317(12)	P(13)-O(37)	1.539(13)	P(18)-O(54)	1.488(12)
Tb(6)-O(47)	2.351(10)	P(13)-O(38)	1.533(12)		
O(1)-Tb(1)-O(4)	79.6(3)	O(5)-Tb(2)-O(16)	78.1(2)	O(13)-Tb(3)-O(22)	77.4(2)
O(1)-Tb(1)-O(7)	101.3(3)	O(5)-Tb(2)-O(19)	155.2(2)	O(13)-Tb(3)-O(23)	115.4(2)
O(1)-Tb(1)-O(8)	169.3(3)	O(8)-Tb(2)-O(10)	79.0(2)	O(13)-Tb(3)-O(25)	77.4(3)
O(1)-Tb(1)-O(20)#1	80.6(3)	O(8)-Tb(2)-O(11)	110.5(3)	O(3)#2-Tb(3)-O(13)	151.3(2)
O(1)-Tb(1)-O(22)#1	93.4(3)	O(8)-Tb(2)-O(13)	141.5(2)	O(17)-Tb(3)-O(19)	77.2(2)
O(1)-Tb(1)-O(24)#1	93.6(3)	O(8)-Tb(2)-O(14)	156.0(3)	O(17)-Tb(3)-O(20)	101.2(3)
O(1)-Tb(1)-O(26)#1	79.2(4)	O(1)-Tb(2)-O(11)	95.5(4)	O(10)-Tb(3)-O(17)	78.0(4)
O(4)-Tb(1)-O(7)	70.7(4)	O(1)-Tb(2)-O(13)	122.8(4)	O(10)-Tb(3)-O(19)	77.8(4)

O(4)-Tb(1)-O(8)	126.2(4)	O(1)-Tb(2)-O(16)	144.4(4)	O(10)-Tb(3)-O(20)	123.5(4)
O(4)-Tb(1)-O(20)#1	154.0(4)	O(2)-Tb(2)-O(5)	103.5(4)	O(10)-Tb(3)-O(22)	154.8(4)
O(4)-Tb(1)-O(22)#1	118.8(4)	O(2)-Tb(2)-O(7)	121.3(4)	O(10)-Tb(3)-O(25)	81.7(4)
O(4)-Tb(1)-O(24)#1	75.4(4)	O(2)-Tb(2)-O(10)	87.7(4)	O(13)-Tb(3)-O(14)	60.4(4)
O(4)-Tb(1)-O(26)#1	78.7(4)	O(2)-Tb(2)-O(11)	84.4(4)	O(13)-Tb(3)-O(17)	72.8(4)
O(7)-Tb(1)-O(8)	76.4(4)	O(2)-Tb(2)-O(13)	79.6(4)	O(13)-Tb(3)-O(19)	95.6(4)
O(7)-Tb(1)-O(20)#1	105.9(4)	O(2)-Tb(2)-O(16)	156.0(4)	O(13)-Tb(3)-O(20)	143.4(4)
O(7)-Tb(1)-O(22)#1	82.2(4)	O(5)-Tb(2)-O(7)	84.2(4)	O(13)-Tb(3)-O(22)	117.6(4)
O(7)-Tb(1)-O(24)#1	146.2(4)	O(5)-Tb(2)-O(10)	140.4(4)	O(13)-Tb(3)-O(25)	142.0(4)
O(7)-Tb(1)-O(26)#1	152.7(4)	O(5)-Tb(2)-O(11)	159.1(4)	O(14)-Tb(3)-O(17)	104.2(4)
O(8)-Tb(1)-O(20)#1	85.9(4)	O(5)-Tb(2)-O(13)	81.5(4)	O(14)-Tb(3)-O(19)	87.3(4)
O(8)-Tb(1)-O(22)#1	59.4(4)	O(5)-Tb(2)-O(16)	85.0(4)	O(14)-Tb(3)-O(20)	91.0(4)
O(8)-Tb(1)-O(24)#1	122.0(4)	O(7)-Tb(2)-O(10)	122.4(4)	O(14)-Tb(3)-O(22)	74.6(4)
O(8)-Tb(1)-O(26)#1	134.9(4)	O(7)-Tb(2)-O(11)	75.3(4)	O(14)-Tb(3)-O(25)	155.6(4)
O(20)#1-Tb(1)-O(22)#1	85.9(4)	O(7)-Tb(2)-O(13)	157.1(4)	O(17)-Tb(3)-O(19)	155.7(4)
O(20)#1-Tb(1)-O(24)#1	147.0(4)	O(7)-Tb(2)-O(16)	81.5(4)	O(17)-Tb(3)-O(20)	140.9(4)
O(22)#1-Tb(1)-O(24)#1	76.5(3)	O(10)-Tb(2)-O(11)	58.0(4)	O(17)-Tb(3)-O(22)	79.8(4)
O(22)#1-Tb(1)-O(26)#1	86.8(3)	O(10)-Tb(2)-O(13)	63.1(4)	O(17)-Tb(3)-O(25)	81.2(4)
O(24)#1-Tb(1)-O(26)#1	81.8(4)	O(10)-Tb(2)-O(16)	72.3(4)	O(19)-Tb(3)-O(20)	58.2(4)
O(20)#1-Tb(1)-O(26)#1	153.6(4)	O(11)-Tb(2)-O(13)	119.2(4)	O(19)-Tb(3)-O(22)	124.2(4)
O(1)-Tb(2)-O(2)	59.0(4)	O(11)-Tb(2)-O(16)	95.6(4)	O(19)-Tb(3)-O(25)	97.3(4)
O(1)-Tb(2)-O(5)	73.1(4)	O(13)-Tb(2)-O(16)	79.6(4)	O(20)-Tb(3)-O(22)	69.7(4)
O(1)-Tb(2)-O(7)	68.9(4)	O(10)-Tb(3)-O(13)	66.5(4)	O(20)-Tb(3)-O(25)	71.7(4)
O(1)-Tb(2)-O(10)	140.7(4)	O(10)-Tb(3)-O(14)	122.7(4)	O(22)-Tb(3)-O(25)	83.2(4)
O(31)-Tb(4)-O(38)	95.9(4)	O(35)-Tb(5)-O(49)	78.8(4)	O(49)-Tb(6)-O(53)	138.9(4)
O(31)-Tb(4)-O(40)	78.6(4)	O(38)-Tb(5)-O(40)	68.1(3)	O(29)#3-Tb(6)-O(49)	139.5(4)
O(31)-Tb(4)-O(53)#2	103.5(4)	O(35)-Tb(5)-O(38)	78.3(4)	O(44)-Tb(6)-O(50)	98.8(4)
O(34)-Tb(4)-O(37)	109.6(4)	O(35)-Tb(5)-O(40)	80.9(4)	O(44)-Tb(6)-O(52)	159.6(4)
O(34)-Tb(4)-O(38)	81.2(4)	O(35)-Tb(5)-O(41)	114.2(4)	O(44)-Tb(6)-O(53)	140.5(4)
O(34)-Tb(4)-O(40)	89.9(4)	O(43)-Tb(5)-O(46)	95.9(4)	O(29)#3-Tb(6)-O(44)	79.8(4)
O(34)-Tb(4)-O(53)#2	87.4(4)	O(43)-Tb(5)-O(47)	72.9(4)	O(32)#3-Tb(6)-O(44)	79.3(4)
O(37)-Tb(4)-O(38)	58.3(4)	O(43)-Tb(5)-O(49)	90.0(4)	O(47)-Tb(6)-O(49)	69.1(3)
O(37)-Tb(4)-O(40)	120.1(4)	O(46)-Tb(5)-O(47)	59.6(3)	O(47)-Tb(6)-O(50)	124.8(4)
O(37)-Tb(4)-O(53)#2	74.8(4)	O(46)-Tb(5)-O(49)	123.5(3)	O(47)#2-Tb(6)-O(52)	78.4(4)
O(38)-Tb(4)-O(40)	70.7(4)	O(47)-Tb(5)-O(49)	69.1(3)	O(47)-Tb(6)-O(53)	114.0(4)
O(38)-Tb(4)-O(53)#2	123.5(4)	O(40)-Tb(5)-O(46)	88.8(3)	O(29)#3-Tb(6)-O(47)	75.6(4)
O(40)-Tb(4)-O(53)#2	164.9(4)	O(40)-Tb(5)-O(47)	135.5(3)	O(32)#3-Tb(6)-O(47)	157.5(4)
O(28)-Tb(4)-O(53)#2	81.5(4)	O(38)-Tb(5)-O(46)	79.5(3)	O(49)-Tb(6)-O(50)	58.6(4)
O(31)-Tb(4)-O(34)#1	168.5(4)	O(38)-Tb(5)-O(47)	127.0(4)	O(29)#3-Tb(6)-O(32)#3	84.8(4)
O(31)-Tb(4)-O(37)	77.7(4)	O(38)-Tb(5)-O(49)	156.1(4)	O(29)#3-Tb(6)-O(50)	159.6(4)
O(28)-Tb(4)-O(31)	89.7(4)	O(40)-Tb(5)-O(41)	58.3(3)	O(32)#3-Tb(6)-O(50)	75.0(4)
O(28)-Tb(4)-O(34)	87.9(4)	O(40)-Tb(5)-O(43)	146.4(4)	O(52)-Tb(6)-O(53)	59.5(4)
O(28)-Tb(4)-O(37)	149.5(4)	O(41)-Tb(5)-O(43)	154.8(4)	O(29)#3-Tb(6)-O(52)	109.1(4)
O(28)-Tb(4)-O(38)	151.8(4)	O(41)-Tb(5)-O(46)	75.8(3)	O(32)#3-Tb(6)-O(49)	124.1(4)

O(28)-Tb(4)-O(40)	83.6(4)	O(40)-Tb(5)-O(49)	114.9(4)	O(50)-Tb(6)-O(52)	79.2(3)
O(38)-Tb(5)-O(41)	120.5(3)	O(41)-Tb(5)-O(49)	75.8(4)	O(50) -Tb(6)-O(53)	96.0(4)
O(38)-Tb(5)-O(43)	80.1(4)	O(41)-Tb(5)-O(47)	82.5(3)	O(32)#3-Tb(6)-O(53)	69.6(4)
O(35)-Tb(5)-O(43)	82.5(4)	O(44)-Tb(6)-O(49)	79.0(4)	O(32)#3-Tb(6)-O(52)	119.1(4)
O(35)-Tb(5)-O(46)	157.7(4)	O(44)-Tb(6)-O(47)	86.5(4)	O(29)#3-Tb(6)-O(53)	74.0(4)
O(35)-Tb(5)-O(47)	139.0(4)	O(49)-Tb(6)-O(52)	82.8(4)		

Symmetry codes: #1, 1-x, -1/2+y, 1-z; #2, -x, -1/2+y, 2-z; #3, -x, 1/2+y, 2-z.

Supplementary Table 5 | Hydrogen bonds among phosphonate groups, $-NH_2-$ groups, $-CH_2-$ groups, phenyl groups, water molecules and NO_3^- anions in ***R*-2**.

D-H···A	*d*(D-H) (Å)	*d*(H···A) (Å)	*d*(D···A) (Å)	∠ DHA (°)
O1W-H1WA···O56	0.85	2.51	2.87(3)	106
O1W-H1WB···O51^{i}	0.85	2.06	2.75(2)	138
O2W-H2WA···O9W^{ii}	0.85	2.21	2.84(2)	131
O2W-H2WB···O60	0.85	2.18	2.82(2)	131
O3W-H3WA···O42^{iii}	0.85	2.31	2.87(2)	124
O3W-H3WB···O59	0.85	2.13	2.75(2)	129
O4W-H4WA···O9	0.85	1.91	2.703(18)	154
O4W-H4WB···O16	0.85	2.30	3.010(18)	142
O5W-H5WA···O23^{iv}	0.85	1.98	2.75(2)	150
O5W-H5WB···O14^{iv}	0.85	2.59	3.25(2)	136
O6W-H6WA···O3	0.85	1.95	2.81(2)	180
O6W-H6WB···O11	0.85	2.60	3.299(19)	141
O7W-H7WA···O21	0.85	2.60	3.227(18)	132
O7W-H7WB···O15	0.85	2.23	2.736(18)	118
O8W-H8WA···O21	0.85	2.37	2.661(19)	100
O8W-H8WB···O60^{v}	0.85	2.56	2.90(2)	106
O9W-H9WA···O21	0.85	2.12	2.894(19)	151
O9W-H9WB···O64	0.85	2.29	2.91(3)	130
O10W-H10O···O66	0.85	2.47	2.93(3)	115
O10W-H10N···O66	0.85	2.58	2.93(3)	106
O11W-H11H···O44^{vi}	0.85	2.52	2.795(19)	100
O11W-H11O···O33	0.85	1.82	2.580(19)	149
O11W-H11N···O1W	0.85	1.97	2.642(19)	136
O12W-H12N···O45^{vii}	0.85	2.38	2.749(19)	107
O12W-H12O···O64	0.85	2.35	2.80(2)	113
N1-H1C···O6	0.99	1.74	2.708(19)	166
N1-H1D···O15	0.99	1.77	2.737(19)	166
N2-H2A···O4W	0.99	1.78	2.765(19)	173
N2-H2B···O8W^{iv}	0.99	2.12	2.81(2)	126
N3-H3D···O5W	0.99	1.92	2.83(2)	152
N3-H3E···O11	0.99	1.89	2.757(19)	145
N4-H4A···O19	0.99	2.01	2.84(2)	140
N4-H4B···O6W	0.99	2.10	2.98(2)	148
N5-H5A···O17	0.99	2.29	3.195(19)	152
N5-H5B···O23	0.99	1.69	2.673(19)	172
N6-H6A···O12	0.99	1.81	2.77(2)	165
N6-H6B···O25	0.99	2.23	3.11(2)	146
N7-H7A···O7W	0.99	1.90	2.848(19)	160
N7-H7B···O8^{v}	0.99	1.89	2.769(18)	146
N8-H8A···O26	0.99	2.03	2.968(19)	158
N8-H8B···O3^{v}	0.99	1.68	2.670(19)	174

N9-H9A···O6^{v}	0.99	2.08	2.928(14)	158
N9-H9B···O62	0.99	1.94	2.87(2)	155
N10-H10J···O45^{vi}	0.99	1.84	2.77(2)	156
N10-H10K···O2W^{viii}	0.99	1.87	2.85(2)	171
N11-H11F···O50^{vi}	0.99	1.74	2.725(19)	172
N11-H11G···O37	0.99	1.72	2.703(19)	172
N12-H12L···O51	0.99	2.07	2.99(2)	154
N12-H12M···O3W^{viii}	0.99	1.94	2.849(14)	151
N13-H13F···O36	0.99	1.87	2.821(19)	159
N13-H13G···O54^{vi}	0.99	1.74	2.712(19)	167
N14-H14F···O46	0.99	1.86	2.786(17)	155
N14-H14G···O31	0.99	1.82	2.747(19)	155
N15-H15F···O12W^{ix}	0.99	1.82	2.80(2)	169
N15-H15G···O36	0.99	1.72	2.70(2)	170
N16-H16A···O43	0.99	1.91	2.885(19)	169
N16-H16B···O39	0.99	1.87	2.803(19)	156
N17-H17A···O52	0.99	1.89	2.708(19)	138
N17-H17B···O41	0.99	1.70	2.679(18)	169
N18-H18A···O48	0.99	1.65	2.623(19)	167
N18-H9B···O53	0.99	2.54	3.082(19)	114
O18-H18E···O57	0.85	1.97	2.57(2)	127
O27-H27A···O63	0.85	1.93	2.607(18)	135
O30-H30F···O59^{v}	0.85	2.00	2.557(18)	122
C9-H9···O61^{iv}	0.95	2.45	3.369(19)	163
C46-H46B···O55	0.99	2.40	3.31(3)	152
C10-H10A···O58	0.99	2.55	3.48(2	156
C161-H161···O65	0.95	2.50	3.35(2)	150

Symmetry transformations used to generate equivalent atoms: i: 1+x,y,-1+z; ii: -x+1, y-1/2, -z+1; iii: x, y, -1+z; iv: 1-x, -1/2+y, 1-z; v: 1-x, 1/2+y, 1-z; vi: -x, -1/2+y, 2-z; vii: 1+x, y, z; viii: x, y, 1+z; ix: -1+x, y, z.

Supplementary Table 6 | Selected bond lengths [Å] and angles [˚] for ***R*-2'**.

Tb(1)-O(1)	2.253(7)	Tb(3)-O(19)	2.448(7)	P(4)-O(12)	1.486(8)
Tb(1)-O(4)	2.381(8)	Tb(3)-O(20)	2.422(8)	P(5)-O(13)	1.539(8)
Tb(1)-O(5)	2.453(7)	Tb(3)-O(22)	2.395(7)	P(5)-O(14)	1.512(8)
Tb(1)-O(7)	2.264(7)	Tb(3)-O(23)	2.563(7)	P(5)-O(15)	1.496(8)
Tb(1)-O(10)	2.269(7)	Tb(3)-O(25)	2.339(8)	P(6)-O(16)	1.504(8)
Tb(1)-O(23)#1	2.295(7)	Tb(3)-O(3)#2	2.289(7)	P(6)-O(17)	1.511(8)
Tb(1)-O(26)#1	2.310(7)	P(1)-O(1)	1.553(8)	P(6)-O(18)	1.536(9)
Tb(2)-O(5)	2.343(7)	P(1)-O(2)	1.481(9)	P(7)-O(19)	1.507(8)
Tb(2)-O(8)	2.265(7)	P(1)-O(3)	1.526(8)	P(7)-O(20)	1.522(8)
Tb(2)-O(10)	2.485(7)	P(2)-O(4)	1.508(8)	P(7)-O(21)	1.474(9)
Tb(2)-O(11)	2.383(8)	P(2)-O(5)	1.531(8)	P(8)-O(22)	1.531(8)
Tb(2)-O(13)	2.476(7)	P(2)-O(6)	1.496(8)	P(8)-O(23)	1.543(7)
Tb(2)-O(14)	2.378(8)	P(3)-O(7)	1.513(8)	P(8)-O(24)	1.479(9)
Tb(2)-O(16)	2.416(7)	P(3)-O(8)	1.515(8)	P(9)-O(25)	1.503(8)
Tb(2)-O(19)	2.326(7)	P(3)-O(9)	1.503(9)	P(9)-O(26)	1.489(8)
Tb(3)-O(13)	2.338(7)	P(4)-O(10)	1.535(8)	P(9)-O(27)	1.543(11)
Tb(3)-O(17)	2.289(7)	P(4)-O(11)	1.557(8)		
O(1)-Tb(1)-O(4)	79.6(3)	O(5)-Tb(2)-O(16)	78.1(2)	O(13)-Tb(3)-O(22)	77.4(2)
O(1)-Tb(1)-O(5)	101.3(3)	O(5)-Tb(2)-O(19)	155.2(2)	O(13)-Tb(3)-O(23)	115.4(2)
O(1)-Tb(1)-O(7)	169.3(3)	O(8)-Tb(2)-O(10)	79.0(2)	O(13)-Tb(3)-O(25)	77.4(3)
O(1)-Tb(1)-O(10)	80.6(3)	O(8)-Tb(2)-O(11)	110.5(3)	O(3)#2-Tb(3)-O(13)	151.3(2)
O(1)-Tb(1)-O(23)#1	93.4(3)	O(8)-Tb(2)-O(13)	141.5(2)	O(17)-Tb(3)-O(19)	77.2(2)
O(1)-Tb(1)-O(26)#1	93.6(3)	O(8)-Tb(2)-O(14)	156.0(3)	O(17)-Tb(3)-O(20)	101.2(3)
O(4)-Tb(1)-O(5)	58.8(2)	O(8)-Tb(2)-O(16)	83.9(3)	O(17)-Tb(3)-O(22)	155.6(3)
O(4)-Tb(1)-O(7)	108.9(3)	O(8)-Tb(2)-O(19)	80.8(3)	O(17)-Tb(3)-O(23)	144.6(2)
O(4)-Tb(1)-O(10)	120.2(3)	O(10)-Tb(2)-O(11)	59.6(2)	O(17)-Tb(3)-O(25)	81.1(3)
O(4)-Tb(1)-O(23)#1	76.9(3)	O(10)-Tb(2)-O(13)	135.8(2)	O(3)#2-Tb(3)-O(17)	75.5(3)
O(4)-Tb(1)-O(26)#1	153.9(3)	O(10)-Tb(2)-O(14)	86.9(2)	O(19)-Tb(3)-O(20)	57.8(2)
O(5)-Tb(1)-O(7)	78.6(3)	O(10)-Tb(2)-O(16)	145.6(2)	O(19)-Tb(3)-O(22)	83.2(2)
O(5)-Tb(1)-O(10)	71.0(2)	O(10)-Tb(2)-O(19)	118.6(2)	O(19)-Tb(3)-O(23)	137.0(2)
O(5)-Tb(1)-O(23)#1	129.1(2)	O(11)-Tb(2)-O(13)	83.9(2)	O(19)-Tb(3)-O(25)	141.6(3)
O(5)-Tb(1)-O(26)#1	147.1(3)	O(11)-Tb(2)-O(14)	77.7(2)	O(3)#2-Tb(3)-O(19)	121.9(3)
O(7)-Tb(1)-O(10)	89.3(3)	O(11)-Tb(2)-O(16)	154.7(2)	O(20)-Tb(3)-O(22)	79.8(3)
O(7)-Tb(1)-O(23)#1	94.9(3)	O(11)-Tb(2)-O(19)	75.2(2)	O(20)-Tb(3)-O(23)	94.1(2)
O(7)-Tb(1)-O(26)#1	81.5(3)	O(13)-Tb(2)-O(14)	59.8(2)	O(20)-Tb(3)-O(25)	159.1(3)
O(10)-Tb(1)-O(23)#1	159.9(3)	O(13)-Tb(2)-O(16)	72.5(2)	O(3)#2-Tb(3)-O(20)	78.9(3)
O(10)-Tb(1)-O(26)#1	82.9(3)	O(13)-Tb(2)-O(19)	68.4(3)	O(22)-Tb(3)-O(23)	58.5(2)
O(23)#1-Tb(1)-O(26)#1	78.4(3)	O(14)-Tb(2)-O(16)	97.6(2)	O(22)-Tb(3)-O(25)	106.8(3)
O(5)-Tb(2)-O(8)	77.7(3)	O(14)-Tb(2)-O(19)	123.1(3)	O(3)#2-Tb(3)-O(22)	128.0(2)
O(5)-Tb(2)-O(10)	69.2(2)	O(16)-Tb(2)-O(19)	87.3(2)	O(23)-Tb(3)-O(25)	73.8(3)
O(5)-Tb(2)-O(11)	124.3(2)	O(13)-Tb(3)-O(17)	82.0(3)	O(3)#2-Tb(3)-O(23)	76.5(2)
O(5)-Tb(2)-O(13)	124.4(3)	O(13)-Tb(3)-O(19)	68.6(3)	O(3)#2-Tb(3)-O(25)	81.6(3)
O(5)-Tb(2)-O(14)	79.2(3)	O(13)-Tb(3)-O(20)	123.6(3)		

Symmetry codes: #1, 2-x, -1/2+y, 3/2-z; #2, 2-x, 1/2+y, 3/2-z.

Supplementary Table 7 | Hydrogen bonds among phosphonate groups, $-NH_2$-groups, $-CH_2$- groups, phenyl groups, water molecules, and NO_3^- anions in ***R*-2'**.

D-H···A	*d*(D-H) (Å)	*d*(H···A) (Å)	*d*(D···A) (Å)	∠ DHA (°)
O1W-H1WA···O12^{i}	0.85	2.16	2.772(12)	129
O1W-H1WB···O26^{ii}	0.85	2.19	2.954(11)	150
O2W-H2WA···O21	0.85	1.98	2.731(13)	147
O2W-H2WB···O31^{iii}	0.85	2.21	2.948(16)	145
N1-H1C···O4	0.99	1.89	2.823(13)	156
N1-H1D···O20^{iv}	0.99	1.76	2.739(12)	169
N2-H2A···O9	0.99	1.89	2.802(13)	152
N2-H2B···O24^{iv}	0.99	1.80	2.775(12)	167
N3-H3D···O8	0.99	2.28	2.721(13)	106
N3-H3E···O1W^{v}	0.99	1.81	2.794(13)	174
N4-H4A···O14	0.99	2.45	2.842(12)	158
N4-H4B···O1	0.99	1.76	2.712(12)	159
N5-H5A···O16	0.99	1.90	2.865(13)	166
N5-H5B···O6	0.99	1.76	2.732(12)	166
N6-H6A···O9	0.99	1.69	2.659(12)	164
N6-H6B···O28	0.99	1.82	2.695(16)	146
N7-H7A···O22	0.99	1.98	2.755(12)	133
N7-H7B···O11	0.99	1.63	2.605(12)	167
N8-H8A···O25	0.99	2.35	3.238(12)	149
N8-H8B···O15	0.99	1.63	2.613(12)	169
N9-H9A···O17	0.99	2.08	2.928(14)	142
N9-H9B···O33^{vi}	0.99	1.95	2.835(15)	147
O18-H18C···O29	0.85	1.90	2.558(14)	133
O27-H27A···O31^{vi}	0.85	1.80	2.581(16)	155
C14-H14···O30	0.95	2.59	3.335(19)	136
C6-H6···O32^{vii}	0.95	2.33	3.23(2)	157

Symmetry transformations used to generate equivalent atoms: i: -1+x,y,z; ii: -x+1, y-1/2, -z+3/2; iii: x+1/2, -y+3/2, -z+2; iv: -x+2, y-1/2, -z+3/2; v: x+1, y, z; vi: -x+1, y+1/2, -z+3/2; vii: -x+1, y+1/2, -z+3/2; viii: 3/2-x,1-y,-1/2+z.

Supplementary Table 8 | Selected bond lengths [Å] and angles [°] for ***R*-3** at 150 K and 277 K.

	**150 K**	**277 K**		**150 K**	**277 K**
Tb(1)-O(1)	2.464(14)	2.463(18)	P(1)-O(1)	1.538(14)	1.530(16)
Tb(1)-O(1)#1	2.338(16)	2.36(2)	P(1)-O(2)	1.508(13)	1.502(16)
Tb(1)-O(2)	2.421(13)	2.442(14)	P(1)-O(3)	1.497(18)	1.497(19)
Tb(1)-O(4)	2.367(18)	2.39(2)	P(2)-O(4)	1.537(17)	1.543(17)
Tb(1)-O(5)	2.632(16)	2.642(19)	P(2)-O(5)	1.542(19)	1.53(2)
Tb(1)-O(5)#2	2.342(15)	2.359(18)	P(2)-O(6)	1.54(2)	1.53(3)
Tb(1)-O(8)	2.28(2)	2.29(2)	P(3)-O(7)	1.532(17)	1.51(2)
Tb(1)-O(7)#1	2.29(2)	2.31(3)	P(3)-O(8)	1.488(17)	1.49(2)
			P(3)-O(9)	1.43(2)	1.44(3)
O(1)-Tb(1)-O5	137.7(5)	137.2(6)	O(5)#2-Tb(1)-O(5)	124.0(7)	124.0(8)
O(1)#1-Tb(1)-O(1)	119.4(5)	119.6(7)	O(8)-Tb(1)-O(1)	74.6(5)	74.2(6)
O(1)#1-Tb(1)-O(2)	76.6(5)	76.7(7)	O(8)-Tb(1)-O(1)#1	77.5(5)	78.2(6)
O(1)#1-Tb(1)-O4	119.9(6)	119.1(6)	O(8)-Tb(1)-O(2)	102.4(6)	101.6(7)
O(1)#1-Tb(1)-O5	65.2(6)	67.8(6)	O(8)-Tb(1)-O(4)	162.2(5)	162.2(5)
O(2)-Tb(1)-O(1)	59.0(4)	58.5(5)	O(8)-Tb(1)-O(5)	139.4(5)	140.0(6)
O(2)-Tb(1)-O5	84.6(5)	84.8(6)	O(8)-Tb(1)-O(5)#2	87.0(7)	87.1(8)
O(4)-Tb(1)-O(1)	92.1(5)	92.1(6)	O(7)#1-Tb(1)-O(1)	145.2(5)	145.8(6)
O(4)-Tb(1)-O(2)	79.8(5)	79.8(6)	O(7)#1-Tb(1)-O(1)#1	84.1(6)	83.3(8)
O(4)-Tb(1)-O(5)	58.1(5)	57.7(6)	O(7)#1-Tb(1)-O(2)	155.6(5)	155.5(6)
O(5)#2-Tb(1)-O(1)	68.0(5)	67.8(6)	O(7)#1-Tb(1)-O(4)	97.5(7)	98.2(8)
O(5)#2-Tb(1)-O(1)#1	159.4(7)	160.1(8)	O(7)#1-Tb(1)-O(5)	73.7(6)	73.9(7)
O(5)#2-Tb(1)-O(2)	120.5(5)	119.8(6)	O(7)#1-Tb(1)-O(5)#2	81.7(5)	82.9(6)
O(5)#2-Tb(1)-O(4)	76.9(7)	77.0(8)	O(7)#1-Tb(1)-O(8)	87.6(7)	87.7(8)

Symmetry code: #1, y, -x+y, z+1/6; #2, x-y, x, z-1/6.

Supplementary Table 9 | Hydrogen bonds among water molecules, phosphonate groups, -NH_2- groups, -CH_2- groups and phenyl groups in ***R*-3** at 150 K.

D-H···A	*d*(D-H) (Å)	*d*(H···A) (Å)	*d*(D···A) (Å)	∠ DHA (°)
N1-H1C···O9	0.99	2.06	3.01(2)	159
N1-H1D···O3	0.99	1.68	2.66(2)	169
N2-H2A···O2	0.99	1.78	2.74(2)	164
N2-H2B···O4	0.99	1.72	2.69(2)	166
N3-H3D···O1W^{i}	0.99	1.93	2.91(5)	166
N3-H3E···O9	0.99	1.93	2.79(4)	145
O1W-H1WC···O7^{ii}	0.85	2.39	2.98(4)	127
O1W-H1WA···O6^{iii}	0.85	2.05	2.71(5)	133
C12-H12C···O3	0.98	2.51	3.44(5)	160
C10-H10B···O1W^{iv}	0.95	2.64	3.52(5)	149

Symmetry transformations used to generate equivalent atoms: i: 1+x, -1+y, -1+z; ii: 1+x, 1+y, 1+z; iii: 1+x-y, 1+x, 5/6+z; iv: -x+y, 1-x, -2/3+z.

Supplementary Table 10 | Cell parameters of ***R*-1** and ***R*-3** obtained by Pawley fitting on the powder X-ray diffraction patterns using Topas 4.2 program.

	***R*-1**	***R*-3**
Cell	$P6_5$	$P6_5$
a (Å)	16.28	15.84
c (Å)	24.24	24.16
V (Å^3)	5564.9	5251.4
R_{wp}	7.2	14.0
$d_{(100)}$ (Å)	14.1	13.7

Supplementary Table 11 | Selected bond lengths [Å] and angles [°] for ***S*-3** at 150 K and 277 K.

	**150 K**	**277 K**		**150 K**	**277 K**
Tb(1)-O(1)	2.471(14)	2.472(14)	P(1)-O(1)	1.532(12)	1.540(14)
Tb(1)-O(1)#1	2.338(15)	2.350(16)	P(1)-O(2)	1.529(13)	1.511(13)
Tb(1)-O(2)	2.422(11)	2.423(13)	P(1)-O(3)	1.500(16)	1.488(17)
Tb(1)-O(4)	2.383(16)	2.390(18)	P(2)-O(4)	1.543(13)	1.528(14)
Tb(1)-O(5)	2.629(14)	2.643(17)	P(2)-O(5)	1.540(18)	1.54(2)
Tb(1)-O(5)#2	2.340(14)	2.341(16)	P(2)-O(6)	1.51(2)	1.54(3)
Tb(1)-O(8)	2.29(2)	2.29(2)	P(3)-O(7)	1.528(17)	1.521(17)
Tb(1)-O(7)#1	2.29(2)	2.31(2)	P(3)-O(8)	1.478(17)	1.481(17)
			P(3)-O(9)	1.40(2)	1.43(2)
O(1)-Tb(1)-O5	137.1(5)	137.5(5)	O(5)#2-Tb(1)-O(5)	124.2(6)	124.5(7)
O(1)#1-Tb(1)-O(1)	118.5(5)	119.3(5)	O(8)-Tb(1)-O(1)	74.9(5)	74.0(5)
O(1)#1-Tb(1)-O(2)	75.9(5)	76.8(5)	O(8)-Tb(1)-O(1)#1	77.3(5)	78.1(5)
O(1)#1-Tb(1)-O4	120.9(5)	119.5(5)	O(8)-Tb(1)-O(2)	103.1(5)	102.2(5)
O(1)#1-Tb(1)-O5	65.6(5)	64.8(6)	O(8)-Tb(1)-O(4)	161.6(5)	162.1(5)
O(2)-Tb(1)-O(1)	59.3(4)	58.7(4)	O(8)-Tb(1)-O(5)	139.4(5)	139.7(5)
O(2)-Tb(1)-O5	83.6(5)	84.5(5)	O(8)-Tb(1)-O(5)#2	87.0(7)	86.8(7)
O(4)-Tb(1)-O(1)	92.2(5)	93.0(5)	O(7)#1-Tb(1)-O(1)	145.8(5)	145.7(5)
O(4)-Tb(1)-O(2)	80.4(5)	80.4(5)	O(7)#1-Tb(1)-O(1)#1	84.2(6)	83.5(6)
O(4)-Tb(1)-O(5)	58.5(5)	57.8(5)	O(7)#1-Tb(1)-O(2)	154.8(5)	155.4(5)
O(5)#2-Tb(1)-O(1)	68.2(4)	67.8(5)	O(7)#1-Tb(1)-O(4)	97.3(7)	96.9(7)
O(5)#2-Tb(1)-O(1)#1	159.7(7)	159.8(7)	O(7)#1-Tb(1)-O(5)	74.1(6)	73.7(6)
O(5)#2-Tb(1)-O(2)	120.7(5)	119.9(5)	O(7)#1-Tb(1)-O(5)#2	82.3(5)	82.7(5)
O(5)#2-Tb(1)-O(4)	76.0(7)	76.8(7)	O(7)#1-Tb(1)-O(8)	87.0(7)	87.8(7)

Symmetry code: #1, y,-x+y,z+1/6; #2, x-y, x, z-1/6

Supplementary References:

1. Liu, Y. et al. Statistical mechanics of helical wormlike chain model. *J. Chem. Phys.* **134**, 065107 (2011).
